# On Information Extraction and Decoding Mechanisms Improved by Noisy Amplification in Signaling Pathways

**DOI:** 10.1038/s41598-019-50631-0

**Published:** 2019-10-07

**Authors:** Aaron Vazquez-Jimenez, Jesus Rodriguez-Gonzalez

**Affiliations:** Centro de Investigación y de Estudios Avanzados del IPN, Unidad Monterrey, Vía del conocimiento 201, Parque de Investigación e Innovación Tecnológica, 66600 Apodaca, NL Mexico

**Keywords:** Computational biophysics, Information theory

## Abstract

The cells need to process information about extracellular stimuli. They encode, transmit and decode the information to elicit an appropriate response. Studies aimed at understanding how such information is decoded in the signaling pathways to generate a specific cellular response have become essential. Eukaryotic cells decode information through two different mechanisms: the feed-forward loop and the promoter affinity. Here, we investigate how these two mechanisms improve information transmission. A detailed comparison is made between the stochastic model of the MAPK/ERK pathway and a stochastic minimal decoding model. The maximal amount of transmittable information was computed. The results suggest that the decoding mechanism of the MAPK/ERK pathway improve the channel capacity because it behaves as a noisy amplifier. We show a positive dependence between the noisy amplification and the amount of information extracted. Additionally, we show that the extrinsic noise can be tuned to improve information transmission. This investigation has revealed that the feed-forward loop and the promoter affinity motifs extract information thanks to processes of amplification and noise addition. Moreover, the channel capacity is enhanced when both decoding mechanisms are coupled. Altogether, these findings suggest novel characteristics in how decoding mechanisms improve information transmission.

## Introduction

The capability of cells to sense, process stimuli, and respond to dynamically fluctuating environments is crucial for survival^1,2^. Cells transduce those stimuli implementing biochemical codes by modulating the dynamical properties as amplitude, frequency, and pulse width of a central molecule^3–6^. Subsequently, such biochemical information is extracted by specific sections of signaling pathways to generate a specific cellular response, this process is known as decoding. A major challenge for cell signaling studies is to understand how different stimuli give rise to specific gene expression responses despite the promiscuous activation of shared pathways^7^. Therefore, cell response depends on how the information is extracted given the intrinsic properties of the decoding mechanisms^1,8–11^.

Eukaryotic cells decode information through two different mechanisms: coherent feedforward loop motif (FFL) and promoter affinity. The FFL is a topological structure commonly found in signaling pathways. This motif consists of three chemical species: a molecule X, which regulates Y, and both jointly regulate molecule Z^12,13^. It is reported that the FFL motif decodes and regulates the inflammatory response in the TLR4 and ERK pathways^2,7,14^. Also, it is proposed that an FFL decodes p53 dynamics to control cell fate^2^. The FFL is named the decoding core just by its temporal properties. However, feedforward loops impose a limit to information processing^6^. Furthermore, FFL is found in numerous functions: the formation of biological memory^15^, persistent stimuli detector^12^, and molecular mechanisms to adaptation^16^.

The second decoding mechanism is based on promoter affinity; it is evidenced on yeast cells. Yeast cells respond to different dynamics of a transcription factor elicited by different stimuli^3,11^. The identities and intensities of different stimuli are transmitted through the modulation of amplitude, duration, or frequency of nuclear translocation factor Msn2 in *Saccharomyces cerevisiae*. Differences in the kinetics of promoter transitions and transcription factor binding properties are reported^17^. Typically, in mammal cells, the signaling pathways are initiated by a stimulus acting on a receptor that is transduced and internalized activating a transcription factor, resulting in the cellular response to the stimulus. Then, the promoter affinity as one decoding structure is suitable in mammals.

Variability permeates biology on all levels, at the cellular level, the molecular interactions are affected by intrinsic and extrinsic fluctuations^18,19^. The stochasticity in those interactions interferes with signal transduction, and it degrades the information transmission^20,21^. On the other hand, negative feedback loops act as a mechanism to noise reduction; they decrease the information by narrowing the dynamic range of response^21^. On yeast and mammal cells, a way to increase information transmission and diminish the noise effects is by having multiple copies of a gene in a cell population^17,21^. Nevertheless, noise properties in information transmission of the decoding mechanisms remain elusive.

Role of FFL motifs during information processing need to be analyzed^6^. Up to now, a systematic study has not been carried out to elucidate the following questions about information transmission in decoding mechanisms: what are the properties of information transmission of the FFL and promoter affinity? What are the advantages of coupling the two decoding mechanisms? Finally, which is the interplay between information transmission and noise sources?

To address this problem, we explored the influence of the decoding mechanisms in information transmission and the role of the intrinsic and extrinsic noise in the signaling pathway. We employ a mechanistic ODE based model of the MAPK/ERK pathway^7^. This pathway participates in the regulation of a large variety of processes, including cell adhesion, cell cycle progression, cell migration, cell survival, differentiation, metabolism, proliferation, and transcription^22^. Two nested FFL motifs are contained in this biochemical network as a decoding core, and it elicits different cellular responses for a particular stimulus^7^. In the current study, we characterized a set of predictions from the stochastic model of the MAPK/ERK pathway and an FFL minimal model to provide insight into the above questions. The properties to information transmission of decoding mechanisms were elucidated applying the information theory as a theoretical background, and the channel capacity (CC) was computed^23^. The CC quantifies the maximal amount of information that is carried and transmitted by the signalling pathway^24–26^. Our results indicate that the amplification and intrinsic noise directly determines the amount of transmitted information on FFL motif. Likewise, by joining both decoding mechanisms, we found that the CC increases. Finally, the system improves information transmission in a noisy environment when the extrinsic fluctuations are controlled. In summary, the characteristics of the decoding mechanisms into information transmission were revealed.

## Results

As previously mentioned, the role of the signaling pathways is to transmit specific information from the extracellular environment to downstream gene expression determining cell fate. Noise due to extrinsic and intrinsic fluctuations associated with biochemical reactions is considered^27^, and it delimits the environmental conditions in which the decoding is held^21,23^. The encoding means how a signaling pathway translate stimuli in the form of dynamics^28^. Purvis and Lahav present an exciting review of experimental evidence that suggests cells can send and receive information by controlling the dynamical properties as amplitude, frequency, and pulse width of their signaling molecules^2^. The MARP/ERK signaling pathway can elicit two different responses depending on the *ppERKcyt* temporal patterns (encode section). The MAPK/ERK pathway transduce different growth factors stimuli (Epidermal and Neural) to induce processes like proliferation and differentiation^22,29^. Both growth factors activate ERK protein by different pathway sections, therefore the stimuli are coded into ERK dynamics.

**Figure 1:**
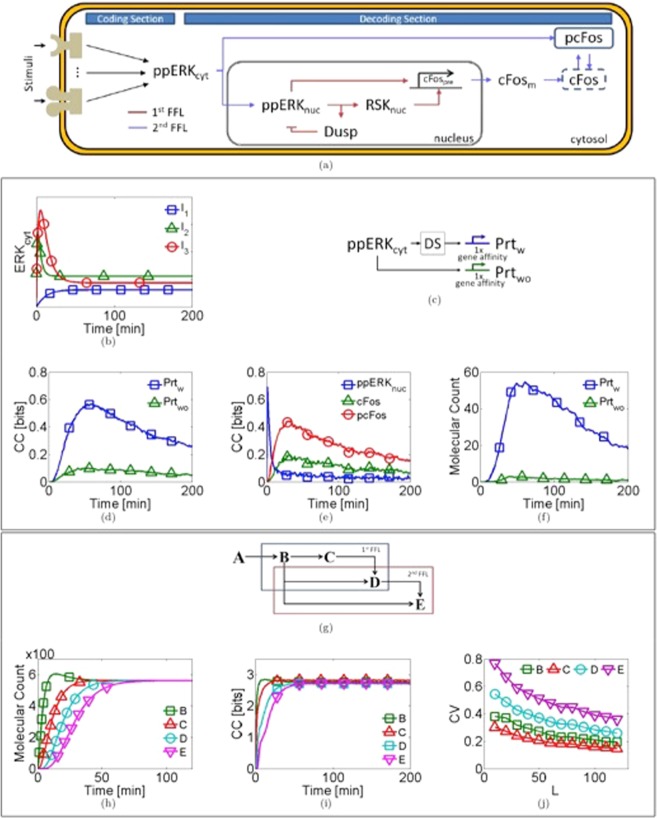
Characterization of the FFL in the information transmission process. (**a**) Decoding section (DS) of the MAPK/ERK pathway structured by two nested coherent type I feedforward loops showed in blue and red arrows. (**b**) Time series of the three used inputs Eq. (18), the parameters for each input are in Table 2, (${\rm{L}}=100$). (**c**) Coupled genes interacting (${{\rm{Prt}}}_{{\rm{w}}}$) and without interacting (${{\rm{Prt}}}_{{\rm{wo}}}$) with the DS of the MAPK/ERK pathway. (**d**) CC versus time for the two coupled genes. (**e**) CC time series for the variables (${{\rm{ppERK}}}_{{\rm{nuc}}}$, ${\rm{cFos}}$, and ${\rm{pcFos}}$) located at the beginning, middle and at the end of the DS. (**f**) Molecular count for proteins ${{\rm{Prt}}}_{{\rm{w}}}$, and ${{\rm{Prt}}}_{{\rm{wo}}}$. (**g**) The minimal model composed of five interacting proteins (B-E) in two nested feedforward loops, under a specific stimulus (${{\rm{A}}={\rm{ppERK}}}_{{\rm{cyt}}}$). (**h**) Time evolution of the minimal decoder model; all the proteins have the same steady-state (${\rm{SS}}$). (**i**) Temporal evolution of the CC for the minimal pathway. (**j**) Coefficient of variation (CV) versus the scale parameter L at ${\rm{t}}=150$ min. For all the variables involved in the minimal model.

The decoding is how a signaling pathway can interpret the encoded stimuli^28^. Complex mechanisms for decoding temporal patterns are based on FFL network motifs. A finely tuned network decodes ERK dynamics, two FFL nested, controlling cell fate decision^7^. ERK is processed solely by one section despite the stimuli identity, in which the information about the stimuli is decoded^30^. Thus, The MAPK/ERK pathway comprises a coding section and a decoding section^1,2^. Decoding section is formed of two nested coherent type I FFL^7^, see Fig. 1(a) (see Supplementary Information for further details about the coding and decoding sections; Fig. S1). The ERK protein corresponds to the input of the decoding section, and the transcription factor pcFos is the output^7^. The pcFos protein activates the downstream gene expression; this process is regulated by the promoter affinity^2,11^. We are interested in study the characteristics of information processing in the decoding section taking into account environmental and molecular fluctuations. Therefore, a stochastic model of the MAPK/ERK pathway was implemented.

To validate the feasibility of the stochastic model, a set of experiments by *Nakakuki et al*. were simulated^7^. In these experiments, the decoding section of the MAPK/ERK pathway was experimentally characterized under three different doses of Epidermal Growth Factor (EGF)^7^ that produce different dynamics of double-phosphorylated ERK cytoplasmic protein (${{\rm{ppERK}}}_{{\rm{cyt}}}$) as input to decode section. The pcFos protein corresponds to the output. We mimicked these experiments by numerically solving the stochastic model for the MAPK/ERK pathway. The corresponding stochastic model and parameters are given in Table 1. The ${{\rm{ppERK}}}_{{\rm{cyt}}}$ dynamics is described by Eq. (18). The different input dynamics were obtained by adjusting the parameters given in Table 2 (See details to simulate inputs in *Modeling the inputs*, *Methods* section). The initial conditions were set to 0 value for all variables. The stochastic model was numerically solved for 200 min to capture the transient response, and the simulation results were compared with the *Nakakuki et al*. experimental data. The system was solved using the Gillespie algorithm, and we carried out over 1000 independent realizations (simulating an isogenic cell population). We report in Fig. 1(b) the results of our stochastic simulations to three different inputs.

**Table 1:** Reactions, propensities and parameter values for the stochastic models increasing ten units at a time.

	Reaction	Propensities	Parameter Value	Description
MAP/ERK pathway model	${{\rm{ppERK}}}_{{\rm{cyt}}}\mathop{\to }\limits^{{{\rm{k}}}_{1}}{{\rm{ppERK}}}_{{\rm{nuc}}}$	${{\rm{k}}}_{1}{{\rm{ppERK}}}_{{\rm{cyt}}}$	15.0 min^−1^	Import of doubly phosphorylated ERK from the cytoplasm to the nucleus
	${{\rm{ppERK}}}_{{\rm{nuc}}}\mathop{\to }\limits^{{\rm{k}}}{\rm{DUSP}}$	${{\rm{kppERK}}}_{{\rm{nuc}}}$	1.0 min^−1^	Production of DUSP
	${{\rm{ppERK}}}_{{\rm{nuc}}}\mathop{\to }\limits^{{{\rm{k}}}_{2}}\rlap{/}{0}$	${{\rm{k}}}_{2}{{\rm{ppERK}}}_{{\rm{nuc}}}$	50.0 min^−1^	Export and dephosphorylation by constitutive phosphatases of doubly phosphorylated ERK
	${{\rm{ppERK}}}_{{\rm{nuc}}}\mathop{\to }\limits^{{k}_{3}{\rm{DUSP}}}\rlap{/}{0}$	${k}_{3}{\mathrm{DUSP}\mathrm{ppERK}}_{{\rm{nuc}}}$	14.0 min^−1^	DUSP-mediated dephosphorylation
	${{\rm{ppERK}}}_{{\rm{nuc}}}\mathop{\to }\limits^{{{\rm{k}}}_{4}}\,{{\rm{pRSK}}}_{{\rm{nuc}}}$	${{\rm{k}}}_{4}{{\rm{ppERK}}}_{{\rm{nuc}}}$	0.1 min^−1^	Activation of the nuclear RSK
	${{\rm{pRSK}}}_{{\rm{nuc}}}\mathop{\to }\limits^{{{\rm{k}}}_{5}}\rlap{/}{0}$	${{\rm{k}}}_{5}{{\rm{pRSK}}}_{{\rm{nuc}}}$	0.15 min^−1^	Dilution and inactivation of the nuclear RSK
	${{\rm{ppERK}}}_{{\rm{nuc}}}+{{\rm{ppRSK}}}_{{\rm{nuc}}}$ $\mathop{\to }\limits^{{\rm{n}},{{\rm{k}}}_{6}}{{\rm{cFos}}}_{{\rm{pre}}}$	$\frac{{({{\rm{ppERK}}}_{{\rm{nuc}}}{\mathrm{pRSK}}_{{\rm{nuc}}})}^{{\rm{n}}}}{{{{\rm{k}}}_{6}}^{{\rm{n}}}+{({{\rm{ppERK}}}_{{\rm{nuc}}}{\mathrm{pRSK}}_{{\rm{nuc}}})}^{{\rm{n}}}}$	${\rm{n}}$=1.1 ${{\rm{k}}}_{6}$=0.13 m^2^	Production of the cFos_pre_ (primary transcript)
	${{\rm{cFos}}}_{{\rm{pre}}}\mathop{\to }\limits^{{{\rm{k}}}_{7}}{{\rm{cFos}}}_{{\rm{m}}}$	${{\rm{k}}}_{7}{{\rm{cfos}}}_{{\rm{pre}}}$	0.5 min^−1^	Processing of pre-mRNA into cFos mRNA
	${{\rm{cFos}}}_{{\rm{m}}}\mathop{\to }\limits^{{{\rm{k}}}_{8}}\rlap{/}{0}$	${{\rm{k}}}_{8}{{\rm{cfos}}}_{{\rm{m}}}$	0.08 min^−1^	mRNA degradation
	${{\rm{cFos}}}_{{\rm{m}}}\mathop{\to }\limits^{{{\rm{k}}}_{9}}{\rm{cFos}}$	${{\rm{k}}}_{9}{{\rm{cfos}}}_{{\rm{m}}}$	0.3 min^−1^	Translation into unphosphorylated and unstable cFos protein
	${\rm{cFos}}\mathop{\to }\limits^{{{\rm{k}}}_{10}}\rlap{/}{0}$	${{\rm{k}}}_{10}{\rm{cFos}}$	0.3 min^−1^	Degradation of the cFos protein
	${{\rm{ppERK}}}_{{\rm{cyt}}}+{\rm{cFos}}$ $\mathop{\to }\limits^{{{\rm{k}}}_{11}}\,{\rm{pcFos}}$	${{\rm{k}}}_{11}{{\rm{ppERK}}}_{{\rm{cyt}}}\,\mathrm{cFos}$	0.11 m^−1^min^−1^	Activation and stabilization of the cFos protein
	${\rm{pcFos}}\mathop{\to }\limits^{{{\rm{k}}}_{12}}\rlap{/}{0}$	${{\rm{k}}}_{12}{\rm{pcFos}}$	0.001 min^−1^	Degradation of the phosphorylated cFos protein
	$\mathrm{pcFos}\,\mathop{\to }\limits^{{{\rm{k}}}_{13}}{\rm{cFos}}$	${{\rm{k}}}_{13}{\rm{pcFos}}$	0.6 min^−1^	Inactivation of the cFos protein
Minimal decoder model	${\rm{A}}\mathop{\to }\limits^{{\rm{k}}}\,B+{\rm{A}}$	${\rm{kA}}$	20 min^−1^	Activation of the protein B
	${\rm{B}}\mathop{\to }\limits^{{\rm{g}}}\rlap{/}{0}$	${\rm{gB}}$	0.1 min^−1^	Dilution and degradation of B
	${\rm{B}}\mathop{\to }\limits^{{\rm{k}}}\,C+{\rm{B}}$	${\rm{kB}}$	20 min^−1^	Activation of the protein B
	${\rm{C}}\mathop{\to }\limits^{{\rm{g}}}\rlap{/}{0}$	${\rm{gC}}$	0.1 min^−1^	Dilution and degradation of C
	${\rm{B}}+{\rm{C}}\mathop{\to }\limits^{{\rm{k}}}\,D+{\rm{B}}+{\rm{C}}$	${\rm{k}}\frac{{\rm{BC}}}{{{\rm{A}}}_{{\rm{N}}}{{{\rm{G}}}_{{\rm{N}}}}^{2}}$	20 min^−1^	Activation of the protein D by B and C
	${\rm{D}}\mathop{\to }\limits^{{\rm{g}}}\rlap{/}{0}$	${\rm{gD}}$	0.1 min^−1^	Dilution and degradation of D
	${\rm{C}}+{\rm{D}}\mathop{\to }\limits^{{\rm{k}}}\,E+{\rm{C}}+{\rm{D}}$	${\rm{k}}\frac{{\rm{CD}}}{{{\rm{A}}}_{{\rm{N}}}{{{\rm{G}}}_{{\rm{N}}}}^{2}}$	20 min^−1^	Activation of the protein E by C and D
	${\rm{E}}\mathop{\to }\limits^{{\rm{g}}}\rlap{/}{0}$	${\rm{gE}}$	0.1 min^−1^	Dilution and degradation of E
Coupled gene model	${\rm{pcFos}}+{{\rm{P}}}_{{\rm{m}}}^{\ast }\mathop{\to }\limits^{{{\rm{\kappa }}}_{{{\rm{P}}}_{{\rm{m}}}},{\rm{n}},{{\rm{K}}}_{{\rm{rp}}}}$ ${{\rm{P}}}_{{\rm{m}}}+\,\mathrm{pcFos}$	${{\rm{\kappa }}}_{{{\rm{p}}}_{{\rm{m}}}}\frac{{{\rm{pcFos}}}^{{{\rm{n}}}_{{\rm{g}}}}}{{{\rm{pcFos}}}^{{{\rm{n}}}_{{\rm{g}}}}+{{\rm{K}}}_{{\rm{rp}}}^{{{\rm{n}}}_{{\rm{g}}}}}{{\rm{P}}}_{{\rm{m}}}^{\ast }$	${{\rm{\kappa }}}_{{{\rm{p}}}_{{\rm{m}}}}$=0.1 min^−1^ ${{\rm{n}}}_{{\rm{g}}}$=2.0 ${{\rm{K}}}_{{\rm{rp}}}$=100 m	Promoter activation
	${{\rm{P}}}_{{\rm{m}}}\mathop{\to }\limits^{{{\rm{\gamma }}}_{{\rm{m}}}}{{\rm{P}}}_{{\rm{m}}}^{\ast }$	${{\rm{\gamma }}}_{{\rm{pm}}}{{\rm{P}}}_{{\rm{m}}}$	0.2 min^−1^	Promoter Inactivation
	${{\rm{P}}}_{{\rm{m}}}\mathop{\to }\limits^{{{\rm{\kappa }}}_{{\rm{m}}}}{\rm{m}}+{{\rm{P}}}_{{\rm{m}}}$	${{\rm{\kappa }}}_{{\rm{m}}}{{\rm{P}}}_{{\rm{m}}}$	14.0 min^−1^	Transcription of the reporter gene
	${\rm{m}}\mathop{\to }\limits^{{{\rm{\gamma }}}_{{\rm{m}}}}\rlap{/}{0}$	${{\rm{\gamma }}}_{{\rm{m}}}{\rm{m}}$	1.0 min^−1^	mRNA degradation
	${\rm{m}}\mathop{\to }\limits^{{{\rm{\kappa }}}_{{\rm{p}}}}{\rm{P}}+{\rm{m}}$	${{\rm{\kappa }}}_{{\rm{p}}}{\rm{m}}$	20.0 min^−1^	Production of the reporter gene
	${\rm{P}}\mathop{\to }\limits^{{{\rm{\gamma }}}_{{\rm{p}}}}\rlap{/}{0}$	${{\rm{\gamma }}}_{{\rm{p}}}{\rm{P}}$	0.1 min^−1^	Protein loss due dilution and degradation

**Table 2:** Numerical parameter values for the inputs.

Parameter	Value			Units
	I_1_	I_2_	I_3_	
k_1_	2.19	1.23	0.257	m*min^−1^
k_2_	1.97	0.949	0.101	m*min^−1^
T_1_	3.16	1.0	9.68	min
T_2_	8.68	3.69	6.3	min

Figure S2 shows our stochastic numerical results together with the experimental results of *Nakakuki et al*. The dynamics of ${{\rm{ppERK}}}_{{\rm{cyt}}}$, ${{\rm{cfos}}}_{{\rm{m}}}$ and pcFos chemical species are shown in Fig S2(a). Figure S2(b) illustrates the results of the deterministic model. Observe that our results match well with *Nakakuki et al*. experimental data. The fact that our stochastic model reproduces the experimental results make us confident that it captures the necessary dynamic characteristics of the MAPK/ERK pathway to attempt further analysis.

### MAPK/ERK pathway model

To evaluate the properties of information transmission of the decoding section in the MAPK/ERK pathway, the channel capacity was computed. It is the maximal amount of information that can be transmitted into a channel^23^. To do this two identical constitutive gene expression systems were coupled: the first one activated through pcFos at the end of the pathway (${{\rm{Prt}}}_{{\rm{w}}}$), and the second was activated directly by ${{\rm{ppERK}}}_{{\rm{nuc}}}$ (${{\rm{Prt}}}_{{\rm{wo}}}$), see Fig.1(c). Each gene expression system is described by Eqs (15–17). The corresponding stochastic model and parameter values are given in Table 1. The initial conditions were set to 0 value for all variables. The stochastic model was numerically solved for 200 min. The probability distribution of each variable, each minute was computed to estimate the CC (see details in *Computation of Channel Capacity*, *Methods section*).

Figure 1(d) illustrates that the gene interacting with the pathway (${{\rm{Prt}}}_{{\rm{w}}}$) has the highest CC value over time. The CC has a transitory temporal dynamic described by three principal characteristics: a rise phase, a maximum value, and a decreasing phase. The CC for three variables (${{\rm{ppERK}}}_{{\rm{nuc}}}$, ${\rm{cFos}}$, and pcFos) is shown in Fig. 1(e). The variables correspond to chemical species positioned downstream at the beginning, middle (output of the first feedforward loop) and the end of the second FFL. As can be observed, the CC increases as the reaction chain progresses. Additionally, the above simulations were repeated for two different inputs (I_1_, I_3_) described in Table 2. Figure S3 shows qualitatively similar results.

This is a counterintuitive result because it is known that in long interaction chains (formed by consecutive reactions) a certain amount of noise is added in every step^27,31^. The noise added leads to the information transmission becomes degraded which implies that the system cannot elicit the proper response. Furthermore, the presence of the feedforward increases the information^21^. The observed information recovery contradicts the data processing inequality; which establishes that the information is lost and never gained when transmitted through a noisy channel^23^. So, the information downstream must be progressively lost at each step^32^. Nevertheless, It is known that the CC can be increased due to noisy amplification processes^31^. Therefore, the ${{\rm{Prt}}}_{{\rm{w}}}$ gene expression system can carry more information. To evaluate the gain dependence in CC, we obtained the molecular dynamics of ${{\rm{Prt}}}_{{\rm{w}}}$ and ${{\rm{Prt}}}_{{\rm{wo}}}$, see Fig. 1(f). It is shown that the expression of the coupled gene at the end of the decoding section is an order of magnitude greater than the other gene. Hence, there is an amplification effect in the decoding section. Figure S4 shows the molecular count for all variables modeled.

Comparing Fig. 1(d,f) shows that the maximum CC value is associated with the ${{\rm{Prt}}}_{{\rm{w}}}$ gene expression system. Notice that the presence of the feedforward loop does not just increase the information capabilities; it increases the gene expression as well. Given the previous results, the following questions were posed: Why then does the decoding section increase the channel capacity rather than decreases it? Is it related to the amplification or the FFL network? To answer these questions, we developed a minimal decoder model, which contains two nested FFL with amplification controlled.

### Minimal decoder model

To analyze the implications of amplification on information gain, we developed a minimal decoder model with normalized amplification, which compared to the MAPK/ERK pathway model is deliberately simplified to capture the essential network characteristics. It was constructed with four proteins (B, C, D, E) interconnected in two nested FFL. The deterministic model is given by Eqs (11–14). The corresponding stochastic model and parameter values are given in Table 1, see Fig. 1(g). The amplification is controlled by the input; it was the same for all proteins (See details in *Minimal decoder mathematical model* section). The input (${\rm{A}}$) was modeled as the input for the MAPK/ERK pathway model, ${{\rm{ppERK}}}_{{\rm{cyt}}}$ (See details to simulate inputs in *Modeling the inputs*, *Methods* section). We repeated the above stochastic simulations. The stochastic model was numerically solved for 200 min. The probability distribution of each variable, each minute was computed to estimate the CC (see details in *Computation of Channel Capacity*, *Methods section*).

Figure 1(h) illustrates the simulation results of the molecular dynamics of B, C, D, and E proteins. As can be observed, the steady-state value (SS) of all proteins is the same. The *CC* for all proteins is reported in Fig. 1(i). The CC for each protein is the same at steady state, ${\rm{CC}}(\infty )$. Therefore, the capacity for transmitting information does not change over the pathway.

To evaluate the noise presence and its propagation into the pathway, we calculated the amount of variability in each chemical species. We adopted the coefficient of variation (CV) to quantify the noise; it is formulated as the standard deviation divided by the mean. Figure 1(j) illustrates the CV for all the proteins in function of the input scale parameter (L). It is evident that as the reaction chain goes forward (${\rm{B}}\to {\rm{E}}$), the variability increases. Consequently, adding variability to the system compensate for the information lost due to the subsequent biochemical reactions. Additionally, the above simulations were repeated for two different inputs (I_1_, I_3_) described in Table 2. Figure S5 shows our results to two additional inputs (I_1_, I_3_) with different steady states. We expected that ${{\rm{CC}}}_{{{\rm{I}}}_{2}}(\infty ) > {{\rm{CC}}}_{{{\rm{I}}}_{3}}(\infty ) > {{\rm{CC}}}_{{{\rm{I}}}_{1}}(\infty )$ because ${{\rm{SS}}}_{{{\rm{I}}}_{2}} > {{\rm{SS}}}_{{{\rm{I}}}_{3}} > {{\rm{SS}}}_{{{\rm{I}}}_{1}}$, see Fig. 1(b). However, our results show that ${{\rm{CC}}}_{{{\rm{I}}}_{3}}(\infty ) > {{\rm{CC}}}_{{{\rm{I}}}_{2}}(\infty ) > {{\rm{CC}}}_{{{\rm{I}}}_{1}}(\infty )$. Interestingly, when we computed the variability induced by the inputs, we found that ${{\rm{CV}}}_{{{\rm{I}}}_{3}} > {{\rm{CV}}}_{{{\rm{I}}}_{2}} > {{\rm{CV}}}_{{{\rm{I}}}_{1}}$(see Fig. S6). These results suggest a positive correlation between CC and amplification and show that the information transmission is enhanced with the addition of intrinsic variability. Furthermore, given the noise addition, what is the advantage of having the FFL instead of a linear interaction chain?

We next developed a comparative study. We compared the minimal decoder model results with a model of four proteins linear interaction chain (see Fig. S7(a,b)). To do that the variables D and E were modified as follows:

$$\frac{{\rm{dD}}}{{\rm{dt}}}={\rm{k}}\frac{{\rm{C}}}{{{\rm{G}}}_{{\rm{N}}}}-{\rm{gD}}$$

$$\frac{{\rm{dE}}}{{\rm{dt}}}={\rm{k}}\frac{{\rm{D}}}{{{\rm{G}}}_{{\rm{N}}}}-{\rm{gE}}.$$

The parameters values are the same for both models. Figure S7(c,d) show that the CC between the two topologies does not change significantly. However, the variability is greater in the last steps of the FFL, see Fig. S7(e,f).

Comparing Fig. 1(h–j), our results suggest that the FFL motif allows the extraction of the maximal amount of information by amplifying and adding noise. It is known that FFL filters and identify stimuli^13,33^. So, the participation of the FFL during the information transmission process is unique compared to a linear interaction chain. Following with the analysis, given that eukaryotic cells decode information through two different mechanisms, what are the advantages of coupling both decoding mechanisms?

### FFL and the promoter affinity mechanisms coupled

**Figure 2:**
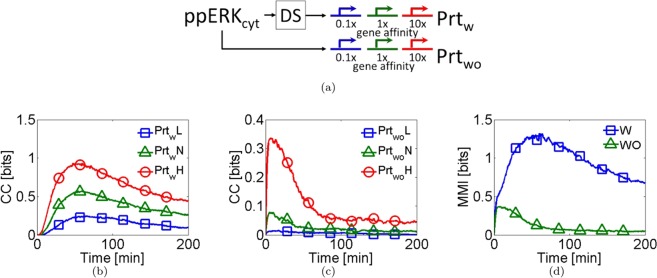
The feedforward loop and the promoter affinity decoding mechanisms coupled. (**a**) Two sets of three genes were coupled with different affinities values. The gene sets were placed interacting (${{\rm{Prt}}}_{{\rm{w}}}$), and the other without interacting (${{\rm{Prt}}}_{{\rm{wo}}}$) with de DS of the MAPK/ERK pathway. The number under the gene indicates the scale factor for the gene affinity given the wild type value. (**b**,**c**) Time series for the CC for both gene sets; the genes with the suffixes **H**, **N**, and **L** are the genes with high, standard and low affinities values respectively. (**d**) Multivariate Mutual Information (MMI) for every gene set over time.

To investigate whether the gene affinity affects the information transmission two sets of three constitutive gene expression systems with different affinities at the beginning and the end of the MAPK/ERK pathway were coupled, see Fig. 2(a). A gene expression system with low affinity (L) was simulated, increasing the parameter value ${{\rm{K}}}_{{\rm{rp}}}$ up to ${10}^{1}$ times its nominal value. The second gene expression system with high affinity (H) was simulated, decreasing ${{\rm{K}}}_{{\rm{rp}}}$ down to ${10}^{-1}$ times its nominal value. The third gene with nominal value was considered (N). The corresponding stochastic model is given in Table 1. We repeated the above stochastic simulations. The stochastic model was numerically solved for 200 min. The probability distribution of each variable, each minute was computed to estimate the CC (see details in *Computation of Channel Capacity*, *Methods section*).

Comparing Fig. 2(b,c), it can be observed that the maximal CC is associated with the genes interacting with the output of the MAP/ERK pathway. Furthermore, notice that our results indicate that the CC is positively correlated to promoter affinity. As in the previous results, the presence of the feedforward loops is advantageous into the information transmission. It is known that the total amount of information is enhanced by considering multiple copies of a gene^21^. Furthermore, it is known that in a clonal cell population, the intrinsic variations can produce differences between the genes, and in their affinity values^18^. So, do multiple genes with different affinity increase the CC as an ensemble?

The total information contained in each gene set was computed. We quantified the total non-redundant information using the multivariate mutual information (MMI), see Fig. 2(d). The gene expression system interacting with the pathway (W) has the highest MMI value compared to the non-interacting gene set (WO). Our results suggest that the heterogeneity in the affinity of a gene ensemble increases the CC. Transcription factors interact at promoters to modulate the transcription of genes. It is known that as the affinity by transcription factors increases, the gene expression increases.

The amplification effect in the CC by considering both decoding mechanisms in a minimal decoder model was analyzed. The CC was calculated for the two sets of three genes added in the minimal decoder model. The affinity values were the same as the ones used for the MAPK/ERK pathway model, see Fig. S8(a). Interestingly, Fig. S8(b) shows that the biggest CC value is associated with the lowest affinity genes. This is opposite to the findings in the MAPK/ERK pathway model. It is known that the variability promotes identifiability. So, the coefficient of variation was calculated (see Fig. S8(c)). Our results suggest that when the gene expression is amplitude limited, the low-affinity genes induced more intrinsic variability, increasing the CC value. The noise addition improves the information transmission process; this is congruent with the previous results.

So far, only the intrinsic variability was considered. It is known that extrinsic noise is present in signaling pathways^31^. Extrinsic noise poses interesting properties that are beneficial to cellular processes^34^. However, under the traditional scheme of information transmission, any noise source is detrimental^21,35,36^. Given the previous results, we pose the following question: How the contributions of extrinsic noise affect the information transmission in the signaling pathways?

### Interplay between the information transmission and the extrinsic noise

**Figure 3:**
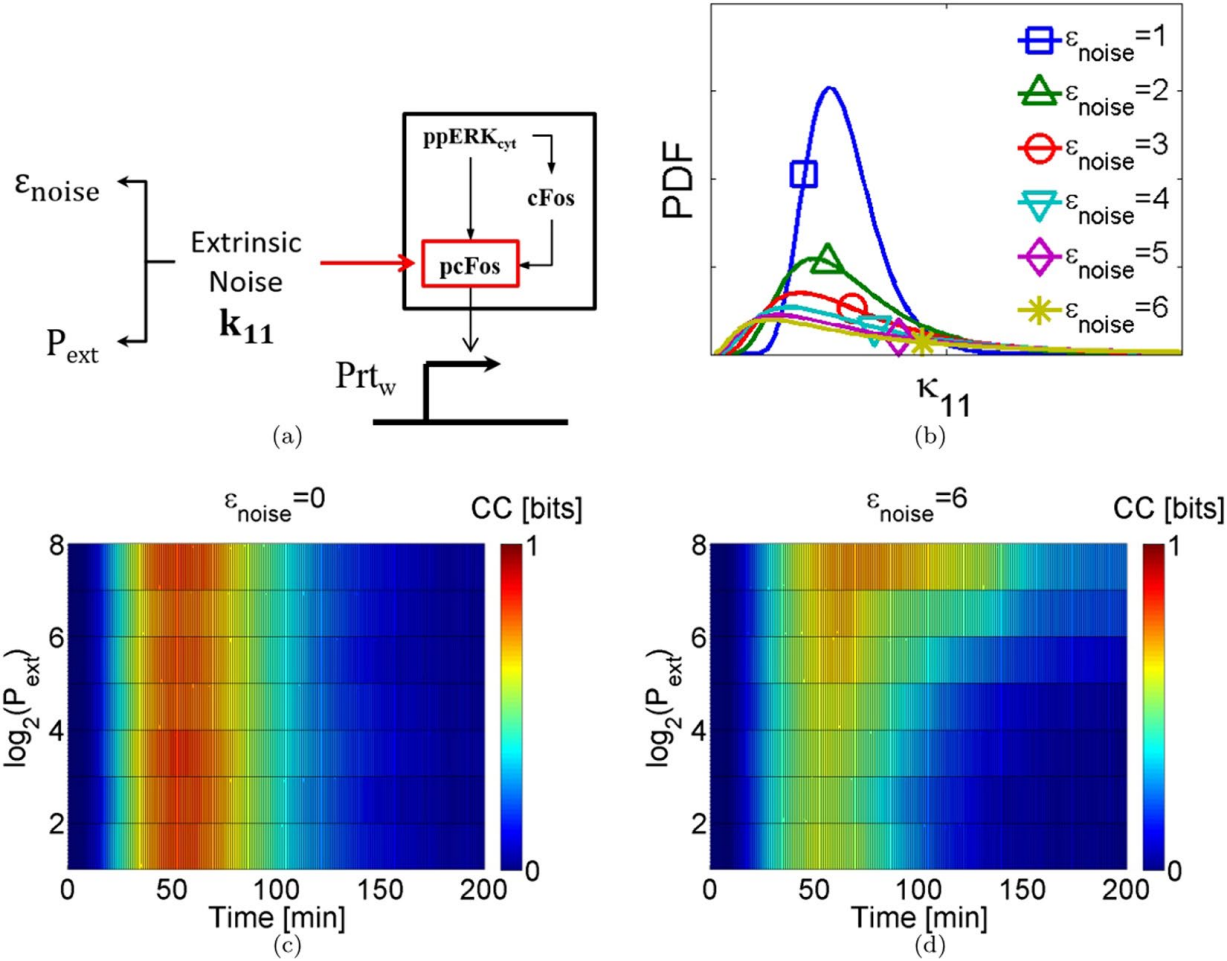
Effect of the extrinsic noise into the information transmission in the MAPK/ERK pathway. (**a**) The extrinsic variability was added to the phosphorylation rate, which is the activation of the transcription factor ${\rm{pcFos}}$; the extrinsic noise was controlled by two parameters: ${{\rm{\varepsilon }}}_{{\rm{noise}}}$ controls the intensity, and ${P}_{{\rm{ext}}}$ is the update frequency. The residence time, it is formulated as the inverse of update frequency. (**b**) The probability distribution function (PDF) under different values of ${{\rm{\varepsilon }}}_{{\rm{noise}}}$. (**c,d**) CC value for the ${{\rm{Prt}}}_{{\rm{w}}}$ protein under the effect of three variables: Time, ${{\rm{\varepsilon }}}_{{\rm{noise}}}$, and ${{\rm{P}}}_{{\rm{ext}}}$. Two extrinsic noise intensities are shown, zero and the highest extrinsic intensity used.

To explore the extrinsic noise effect into the cellular information transmission, stochastic simulations were carried out with an extrinsic noise source controlled. Following to Ladbury *et al*. and Huh *et al*.^27,37^, we chose a cell machinery component associated with the phosphorylation$,{k}_{11}$ (the phosphorylation of pcFos), as the random variable that represents extrinsic noise source. We modified the algorithm to take control of the extrinsic noise source, see Fig. 3(a). Two parameters to control the extrinsic noise source were introduced: the intensity $({{\rm{\varepsilon }}}_{{\rm{noise}}}$) and the update period (${{\rm{P}}}_{{\rm{ext}}}$). ${{\rm{\varepsilon }}}_{{\rm{noise}}}$ corresponds to the shape of the lognormal probability distribution and ${{\rm{P}}}_{{\rm{ext}}}$ represents the period with which ${{\rm{k}}}_{11}$ was updated (see Fig. 3(b)). We repeated the above stochastic simulations. The stochastic model was numerically solved for 200 min. The probability distribution of each variable; each minute was calculated; the CC was computed to different ${{\rm{\varepsilon }}}_{{\rm{noise}}}{\mathrm{and}P}_{{\rm{ext}}}$ values. (see Modeling extrinsic noise, Methods Section).

Figure 3(c,d) illustrate the CC of the protein-coupled at the end of the pathway to eight ${{\rm{P}}}_{{\rm{ext}}}\,$values and two extrinsic noise intensities. The color bar represents the CC value. In Fig. 3(c), the CC remains constant in function of ${{\rm{P}}}_{{\rm{ext}}}$ because ${{\rm{\varepsilon }}}_{{\rm{noise}}}$ is zero; it corresponds to control experiment. Under noisy conditions, the CC value decreases as ${{\rm{\varepsilon }}}_{{\rm{noise}}}$ increases. Then, the extrinsic noise is detrimental to the information transmission process, see Fig. 3(d). However, as the update period increases (low-frequency noise), the CC increases. Our simulations results suggest that the system could handle high noise intensity with low update period to transmit information with more accuracy. Therefore, the impact of the extrinsic noise can be buffered. Similar results are obtained for different ${{\rm{\varepsilon }}}_{{\rm{noise}}}$ values (see Fig. S9).

## Discussion

Cells interact with their environment surrounded by a plethora of stimuli. Each stimulus is information which is sent with the intent of eliciting a response. Within cells, the information is encoded, transmitted, and decoded via signaling pathways, and it is affected by molecular noise. The feedforward loop and the promoter affinity are common motifs as decoding mechanisms and are present in different signaling pathways^2^. A major challenge for cell signaling studies is to understand information transmission properties in decoding mechanisms. To do that, we have used Shannon’s information theory that provides a mathematical framework to quantify the amount of information to be transmitted in a noisy biochemical channel^23^.

To elucidate the characteristics to transfer information of the decoding section that contains FFL in their structure; we investigated two stochastic models: the MAPK/ERK pathway model, and a minimal decoder model. The decoding section of the MAPK/ERK pathway contains a nested double-feedforward loop. It can elicit different responses (i.e., cellular differentiation and proliferation) when it is stimulated with two different dynamics (same chemical entity)^7^. Our results reveal that the FFL increases the CC (see Fig. 1(d)–(f) and Fig. S3). Moreover, the FFL increases the molecular count in middle reactions of decoding section (see Fig. S4). The gene with higher expression carried more information than gene expression systems activated directly by ${{\rm{ppERK}}}_{{\rm{nuc}}}$. According to the data processing inequality, the information is lost when it is transmitted through a noisy channel^23^. Additionally, it is known that the FFL restricts the amount of information due to the way is interconnected; it promotes the information lost^6^.

To understand why our predictions are not in agreement with the data processing inequality, we studied if the effect is due to FFL motive or amplification. A minimal model was designed. The minimal model has the same FFL nested topology as the MAP/ERK pathway model with controlled amplification. Our results show that 1) the capacity for transmitting information does not change along the pathway, see Fig. 1(i). 2)The noise increases as the reaction chain goes forward. The same results are observed for the inputs I_1_ and I_3_ (see Figs S5, 6). As can be seen, there is an interplay between the amplification and noise; both are necessary to increase the amount of transmittable information. Based on the MAP/ERK and minimal model predictions, our results indicate that the decoding section has the property of amplifying and adding noise. These characteristics would allow sending information with a low probability of error given external stimuli, and the cell could, for example, proliferate or differentiate.

Recall that under noiseless transmission, the probability distribution of the elicited responses for a specific input is a Dirac delta. The noise induces responses with wider distributions, reducing the response identifiability due to the distributions overlapping. However, it is known that as the width of the distributions increases the dynamic range increases; so, it can lead to better identifiability increasing the CC value^21,38^. Ideally, every stimulus induces a unique response distribution on a molecule. Nonetheless, when the response distributions by two or more stimuli are close to each other, there is an overlapping area. In this area, the molecule has the same expression for different inputs, leading to elicit a random outcome given the stimuli. By making the response distribution wider, it increases the possible expression values that the molecule can take, even if there is an overlapping area, there are more unique expression values to trigger the correct response. Then, the identifiability is related to the dynamic range, which is directly affected by the shape of the responses distribution^26,39^, and the shape is determined by the variability induced by the noise level. Afterward, intrinsic variability is beneficial to the information transmission process.

Nonetheless, it is stated that the noise decreases the transmission quality^21,35,36^. Experimental^21^ and theoretically^32^ reports establish that the motifs for preventing noise propagation cause a greater information loss. These motifs restrict the information capacity (i.e., negative feedback loops). So, the cell could have developed a manner to transmit information by using the noise as an enhancer. Even though we associated the amplification as the main process to increase the capabilities to transmit information, the intrinsic noise helps. Additionally, it may be seen that comparing the minimal decoder model with a linear interaction chain, the CC between the two pathways does not change significantly. However, the variability increases in the latest steps in the pathway with the feedforward loop in contrast to the linear interaction pathway, see Fig. S7.

According to Tikhonov *et al*.^40^, the relevant quantity is not the amount of total information; the relevant quantity is the accessible information (the amount of information the downstream target access). It has been established theoretically and experimentally a difference between these two types of information. Although the total information decreases in every step according to the information processing inequality, the accessible information can be increased in every step. This can be produced through noisy amplification and balanced response times. So, multi-tier reaction chains are better to extract information if a noisy amplification process is involved. This agrees with our findings (see Fig. 1). We have observed that the noisy amplification controls the amount of extracted information. Overall, our results suggest that the FFL is a noisy amplifier.

Our next task was to investigate the influence of the second decoding mechanism, the promoter affinity. This mechanism has been experimental and theoretically studied on yeast cells^3,11,17^. We explored the relationship between promoter affinity and information transmission. Two sets of three genes with different affinities were coupled, see Fig. 2(a). Our results show that the CC increases as the affinity increases, see Fig. 2(b,c).

Considering that in a clonal population, the intrinsic variability produces promoter affinity differences, we evaluated the MMI (the total non-redundant information) in the two gene sets. The MMI was bigger for both gene sets in contrast to their individual contributions, see Fig. 2(d). Our results are in agreement with Cheong *et al*.^21^. They have demonstrated that multiple copies of a gene effectively increase the gained information in the NF-κB signaling pathways. Cells discriminate between many TNF concentrations. A gene ensemble extracts more information than a single gene. Our current findings expand this idea, in an isogenic population exists intrinsic differences between the genes^41^. One of these differences corresponds to affinity by their transcription factors. When the non-redundant information is computed, it increases in a gene ensemble. However, the non-redundant information is not the addition of the total information extracted by every gene. That means that every gene extracts an amount of unique information. Moreover, the genes extract redundant information, which brings robustness to the system^42^. Although the use of signaling pathways is associated with the ability to differentiate diverse stimuli eliciting different responses^2^, signaling pathways cannot discriminate intensities. On yeast, it has been demonstrated that the affinity helps to differentiate only the stimulus identity and not the intensity; they behave like a switch^17^. Under this paradigm, the decoding mechanism performs binary decisions. Biologically, our results allow us to establish new experiments about how a clonal cell population could improve their response given external stimuli under maximum MMI conditions.

Finally, we investigated the contribution of the extrinsic noise in information transmission. The previous results considered only the intrinsic variability due to molecular interactions. Nevertheless, the molecular interactions in cells are affected by intrinsic and extrinsic fluctuations. They are produced by the promiscuity of the protein-protein interaction resulting from the pathways cross-talk^43^. To explore the effect of the extrinsic noise, we considered variability in the phosphorylation of ${\rm{pcFos}}$^37,43^. Our results showed that the CC value decreases as the noise intensity increases. Therefore, the extrinsic noise is detrimental to the information transmission process (see Fig. 3(d)). However, the system can handle high-intensity noise at low-frequency. Comparable results are obtained for different intensities values, see Fig. S9. Our results further suggest that systems under low-frequency noise, no matter the intensity, enhance the information transmission.

According to Selimkhanov *et al*.^44^, the extrinsic noise increases the information transfer by increasing the dynamic range of the response. Nevertheless, the information dramatically diminished when extrinsic and intrinsic noise sources -just as was done in the present study- are considered. The intrinsic noise adds uncorrelated uncertainty; in contrast, the extrinsic variability produces fluctuations constrained to network dynamic^18,44^. Our results are in agreement with these studies. Furthermore, Selimkhanov *et al*. showed that dynamic responses maximize information accuracy. Additionally, the negative feedback loops provide robustness to the pathway diminishing the extrinsic noise; it protects the information transferred^36^. Lestas *et al*.^32^ demonstrated that the transmission of chemical information is not fundamentally limited by the number of molecules but by the number of chemical events integrated over the timescale. They established that the error of transmission decreases as the fourth root of the number of events, which is too energetically costly. Our results suggest that instead of increasing the number of steps, the cells could develop complex interaction motifs (feedforward loops) to improve information transmission. Besides, if the system is under low-frequency noise, the information transmission is enhanced. Additionally, under controlled laboratory conditions, our results predict conditions under which cells could allow sending information with a low probability of error given external stimuli in a noisy environment. Cells could, for example, proliferate or differentiate.

This study opens several lines of research worth pursuing in future work. It would be worthwhile to extend a similar investigation considering the information carried into the temporal dynamics of the response. It is still unclear the relationship between the information contained in the dynamical patterns and the decoding mechanisms properties to extract the information. This implies the development of theoretical tools to perform the quantification. As well, there are different metrics defined in information theory that can be used to characterize the properties and limitations of signaling pathways, for example, the rate-distortion function. Additionally, the cells use different coding schemes; in the present work, the scheme considered was a mixture between the amplitude and the pulse-width modulation. Moreover, the frequency modulation is used by the MAP/ERK and calcium pathways to code information. However, there is a lack of information about the efficiency properties and limitations of the different encoding schemes. Furthermore, it would be useful to considerer explicitly the complete modulation-demodulation process, and it would be suitable to study the multiple extrinsic noise sources (i.e., extrinsic noise in the gene expression) to evaluate the interplay between the information transmission and noise.

## Conclusion

We can conclude from these facts that the characteristics of two nested coherent type I FFL and the promoter affinity as decoding mechanisms of the MAPK/ERK pathway to information transmission are coupling, amplifying and adding variability increasing the achievable information. The achievable information is improved when both mechanisms are coupled. We demonstrated that an FFL well-tuned under low-frequency extrinsic noise enhances the information transmission. Finally, the stochasticity inherent in a cell population is essential to produce differences in the promoter affinity and increase the information transmission capabilities.

## Methods

**Figure 4:**
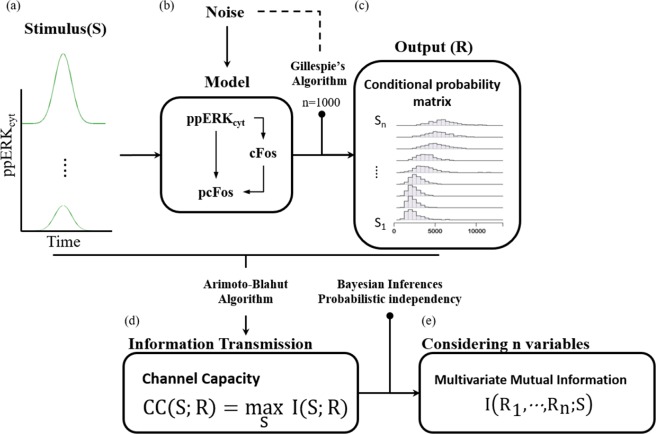
Algorithm for estimating CC and MMI. (**a**) Inputs definition are given in Table 2, the input was scaled from ${\rm{L}}=10$ through ${\rm{L}}=120$, by increasing the value ten units at a time. (**b**) Stochastic models of the MAPK/ERK pathway or minimal decoder is selected. The propensities and parameter values are given in Table 1. (**c**) Estimation of the response conditional probability distribution for every simulation minute. (**d**,**e**) Channel Capacity or Multivariate Mutual Information is computed.

The information transmitted in the cell is decoded and interpreted by specific signaling pathways to generate a cellular response. The algorithm depicted in Fig. 4 was used to study the characteristics of the information transmission process and to compute the channel capacity. Algorithm steps: 1) The input is defined. 2) A stochastic model is selected between two options: the MAPK/ERK pathway model or the minimal decoder model. 3) The conditional probability distributions are estimated. 4) the information theory metrics are computed. They are explained below.

### MAPK/ERK pathway mathematical model

Mitogen-activated protein kinase (MAPK) signal transduction pathways are among the most widespread mechanisms of eukaryotic cell regulation. All eukaryotic cells possess multiple MAPK pathways that are activated by a distinct set of stimuli, allowing the cells to respond to multiple and different inputs^45^. Particularly, MAPK/ERK pathway is identified on mammals and coordinates diverse cellular activities like proliferation, differentiation, gene expression, cellular metabolism, motility, survival and apoptosis^22,46^. Pathway activation begins with the recognition of growth factors by their surface receptors to elicit a phosphorylation chain to activate double phosphorylated Extracellular signal–Regulated Kinases (${{\rm{ppERK}}}_{{\rm{cyt}}}$). ${{\rm{ppERK}}}_{{\rm{cyt}}}$ is accumulated in the nucleus to phosphorylate several transcription factors. Is been proved that dysregulation of this pathway can induce cancer and congenital disorders^47,48^.

MAPK/ERK pathway is divided into two sections: coding and decoding. Nakakuki *et al*.^7^ characterized the decoding section combining mathematical modeling and experimental results. They described the temporal evolution of all chemical species depicted in Fig. 1(a) with a deterministic mathematical model of ten differential Eqs (1)–(10). The decoding section input corresponds to the activated Extracellular signal–Regulated Kinases (${{\rm{ppERK}}}_{{\rm{cyt}}}$); it was mathematically modeled to several stimuli corresponding to different growth factors. ${{\rm{ppERK}}}_{{\rm{cyt}}}$ is the output of the coding section (see Fig. S1). ${{\rm{ppERK}}}_{{\rm{cyt}}}$ is internalized into the nucleus (${{\rm{ppERK}}}_{{\rm{nuc}}}$) with a rate ${{\rm{k}}}_{1}$. ${{\rm{ppERK}}}_{{\rm{nuc}}}$ is dephosphorylated by constitutive phosphatases and by Dual Specific Phosphatase (${\rm{DUSP}}$) with rates ${{\rm{k}}}_{2}$ and ${{\rm{k}}}_{3}$, respectively. DUSP is synthetized with a rate k. The nuclear internalization of ${\rm{ppERK}}$ linearly activates the kinase (${{\rm{pRSK}}}_{{\rm{nuc}}}$) with a rate ${k}_{4}$; it is degraded by constitutive phosphatases with a rate ${{\rm{k}}}_{5}$. ${{\rm{ppERK}}}_{{\rm{nuc}}}$ and ${{\rm{pRSK}}}_{{\rm{nuc}}}$ activate the transcription of (${{\rm{cfos}}}_{{\rm{pre}}}$). It is a functional transcription factor of the pathway. The production of primary transcript ${{\rm{cfos}}}_{{\rm{pre}}}$ is modeled with a hill function (parameters: ${{\rm{k}}}_{6}$ and ${\rm{n}}$), and processed with a rate ${{\rm{k}}}_{7}$ into mature mRNA, (${{\rm{cfos}}}_{{\rm{m}}}$). It is degraded at a rate ${{\rm{k}}}_{8}$. ${{\rm{cfos}}}_{{\rm{m}}}$ is translated into un-phosphorylated and unstable (${\rm{cFos}})$ protein with a rate ${{\rm{k}}}_{9}$. The protein is degraded with linear kinetics with a rate ${{\rm{k}}}_{10}$. To be functional, ${\rm{cFos}}$ needs to be stabilized by ${{\rm{ppERK}}}_{{\rm{cyt}}}$; it is done with a rate ${{\rm{k}}}_{11}$ (pcFos). However, this process is reversible by the interaction of phosphatases at a rate ${{\rm{k}}}_{13}$. Finally, the protein pcFos is degraded with first-order kinetics at a rate ${{\rm{k}}}_{12}$.

Topologically, the decoding section is structured in two nested coherent FFL. The inner FFL is present at the transcription of ${\rm{cFos}}$ and the second one at the stabilization and phosphorylation of the same protein. The deterministic mathematical model is available from the Biomodels database under the ID 1003170000 (http://www.ebi.ac.uk/biomodels/), and it is given by:

1 $${{\rm{ppERK}}}_{{\rm{cyt}}}={{\rm{x}}}_{1}-{{\rm{x}}}_{2}$$

2 $$\frac{{{\rm{dppERK}}}_{{\rm{nuc}}}}{{\rm{dt}}}={{\rm{k}}}_{1}{{\rm{ppERK}}}_{{\rm{cyt}}}-{{\rm{ppERK}}}_{{\rm{nuc}}}({{\rm{k}}}_{2}+{{\rm{k}}}_{3}{\rm{Dusp}})$$

3 $$\frac{{{\rm{dpRSK}}}_{{\rm{nuc}}}}{{\rm{dt}}}={{\rm{k}}}_{4}{{\rm{ppERK}}}_{{\rm{nuc}}}-{{\rm{k}}}_{5}{{\rm{pRSK}}}_{{\rm{nuc}}}$$

4 $$\frac{{{\rm{dcfos}}}_{{\rm{pre}}}}{{\rm{dt}}}=\frac{{({{\rm{ppERK}}}_{{\rm{nuc}}}{\mathrm{pRSK}}_{{\rm{nuc}}})}^{{\rm{n}}}}{{({{\rm{ppERK}}}_{{\rm{nuc}}}{\mathrm{pRSK}}_{{\rm{nuc}}})}^{{\rm{n}}}+{{{\rm{k}}}_{6}}^{{\rm{n}}}}-{{\rm{k}}}_{7}{{\rm{cfos}}}_{{\rm{pre}}}$$

5 $$\frac{{{\rm{dcfos}}}_{{\rm{m}}}}{{\rm{dt}}}={{\rm{k}}}_{7}{{\rm{cfos}}}_{{\rm{pre}}}-{{\rm{k}}}_{8}{{\rm{cfos}}}_{{\rm{m}}}$$

6 $$\frac{{\rm{dcFos}}}{{\rm{dt}}}={{\rm{k}}}_{9}{{\rm{cFos}}}_{{\rm{m}}}+{{\rm{k}}}_{13}{\rm{pcFos}}-{\rm{cFos}}({{\rm{k}}}_{10}+{{\rm{k}}}_{11}{{\rm{ppERK}}}_{{\rm{cyt}}})$$

7 $$\frac{{\rm{dDUSP}}}{{\rm{dt}}}={\rm{kDUSP}}$$

8 $$\frac{{\rm{dpcFos}}}{{\rm{dt}}}={{\rm{k}}}_{11}{\rm{cFos}}\,{{\rm{ppERk}}}_{{\rm{cyt}}}-{\rm{pcFos}}({{\rm{k}}}_{12}+{{\rm{k}}}_{13})$$

9 $$\frac{{{\rm{dx}}}_{1}}{{\rm{dt}}}=\frac{-{{\rm{x}}}_{1}+{{\rm{K}}}_{1}{\rm{L}}}{{{\rm{\tau }}}_{1}}$$

10 $$\frac{{{\rm{dx}}}_{2}}{{\rm{dt}}}=\frac{-{{\rm{x}}}_{2}+{{\rm{K}}}_{2}{\rm{L}}}{{{\rm{\tau }}}_{2}}.$$

A stochastic simulation algorithm (SSA) was implemented to study the noise effect in information transmission of the MAPK/ERK decoding section. The reactions with their effective propensities and parameter values are tabulated in Table 1.

### Minimal decoder mathematical model

The aim of designing a minimal decoder mathematical model was to explore the relationship between the amplification and the information transmission capacity into the FFL. To do so, we developed a model that lacks amplification *per se*. The amplification was controlled by the dynamical properties of the input. The model considered the topological characteristics of the decoding section of the MAPK/ERK pathway. The model was composed by the stochastic interaction of four proteins (B–E) interconnected in two nested FFL, see Fig. 1(g). The number of parameters was reduced, we assumed that all the proteins were equal. The equations for the B, C, D and E proteins are given by:

11 $$\frac{{\rm{dB}}}{{\rm{dt}}}={\rm{kA}}({\rm{t}})-{\rm{gB}}$$

12 $$\frac{{\rm{dC}}}{{\rm{dt}}}={\rm{k}}\frac{{\rm{B}}}{{G}_{N}}-{\rm{gC}}$$

13 $$\frac{{\rm{dD}}}{{\rm{dt}}}={\rm{k}}\frac{{\rm{BC}}}{{{\rm{A}}}_{{\rm{N}}}{{{\rm{G}}}_{{\rm{N}}}}^{2}}-{\rm{gD}}$$

14 $$\frac{{\rm{dE}}}{{\rm{dt}}}={\rm{k}}\frac{{\rm{CD}}}{{{\rm{A}}}_{{\rm{N}}}{{{\rm{G}}}_{{\rm{N}}}}^{2}}-{\rm{gE}}.$$

This model consists of four differential Eqs (11)–(14) that respectively account for the temporal evolution of all chemical species concentrations depicted in Fig. 1(g). The parameters ${\rm{k}}$ and ${\rm{g}}$ indicate the production and degradation rates, respectively. The minimal model was evaluated under same inputs as the MAP/ERK model. Therefore ${\rm{A}}({\rm{t}})={{\rm{ppERK}}}_{{\rm{cyt}}}({\rm{t}})$, it is described by Eq. (18). To differentiate the effect in the information transmission due to the amplification and the feedforward, all the modeled proteins have a normalized steady-state. It was controlled by using two parameters ${{\rm{A}}}_{{\rm{N}}}={\rm{L}}({{\rm{k}}}_{1}-{{\rm{k}}}_{2})$ and ${{\rm{G}}}_{{\rm{N}}}={\rm{k}}/{\rm{g}}$. The steady state for all the variables was ${\rm{SS}}={{\rm{A}}}_{{\rm{N}}}{{\rm{G}}}_{{\rm{N}}}$. A SSA was implemented to study the noise effect in information transmission of the minimal pathway section. The reactions, propensities and parameter values are given in Table 1.

### Coupled gene model

The reporter genes were placed interacting at different levels of the pathway. The constitutive gene expression system was modeled by three differential equations:

15 $$\frac{{{\rm{dP}}}_{{\rm{m}}}}{{\rm{dt}}}=\frac{{{\rm{TF}}}^{{\rm{n}}}}{{{\rm{TF}}}^{{\rm{n}}}+{{\rm{K}}}_{{\rm{rp}}}^{{\rm{n}}}}(1-{{\rm{P}}}_{{\rm{m}}})-{{\rm{\gamma }}}_{{\rm{pm}}}{{\rm{P}}}_{{\rm{m}}}$$

16 $$\frac{{\rm{dm}}}{{\rm{dt}}}={{\rm{\kappa }}}_{{\rm{m}}}{{\rm{P}}}_{{\rm{m}}}-{{\rm{\gamma }}}_{{\rm{m}}}{\rm{m}}$$

17 $$\frac{{\rm{dP}}}{{\rm{dt}}}={{\rm{\kappa }}}_{{\rm{p}}}{\rm{m}}-{{\rm{\gamma }}}_{{\rm{p}}}{\rm{P}}$$

This model account for the temporal evolution of the promotor activated (P_m_), and the concentrations of mRNA (m) and proteins (P). Parameters ${{\rm{\kappa }}}_{{\rm{m}},}$ and ${{\rm{\kappa }}}_{{\rm{p}}}$ represent the mRNA and protein production rates, respectively. Parameters ${{\rm{\gamma }}}_{m}$and ${{\rm{\gamma }}}_{{\rm{p}}}$denote the mRNA and protein degradation rates. The promoter dynamic was described as a Hill function, where n is the Hill constant and ${{\rm{K}}}_{{\rm{rp}}}$ is the inverse of gene affinity for the transcription factor, *TF*. The stochastic simulation was carried out using SSA. The reactions with their effective propensities and parameter values are tabulated in Table 1.

### Modeling the inputs

Nakakuki *et al*.^7^ characterized phenomenologically the dynamics of ppERK_cyt_ for three doses of epidermal growth factor (0.1 nM, 1 nM, and 10 nM). ppERK_cyt_ is mathematically described by the Eqs (1), (9)–(10). The solution is given by Eq. (18); the different temporal traces were obtained by adjusting the parameters given in Table 2. They are presented in Fig. 1(b). The input must satisfy that ppERK_cyt_(t) ≥ 0; therefore, k_1_ > k_2_ and all parameters are positive.

18 $${{\rm{ppERK}}}_{{\rm{cyt}}}({\rm{t}})={\rm{L}}[{{\rm{k}}}_{2}({{\rm{e}}}^{-\frac{{\rm{t}}}{{{\rm{T}}}_{2}}}-1)-{{\rm{k}}}_{1}({{\rm{e}}}^{-\frac{{\rm{t}}}{{{\rm{T}}}_{1}}}-1)]$$

To set the input for our experiments, we fixed the parameters and change the amplification, L. It is the scale parameter to control the amplification. We increased the value of parameter L from 10 to 120, by increasing ten units at a time.

We calculated the derivate of Eq. (18), to stochastically model the dynamics of the input ppERK_cyt_. Two terms equation with the opposite sign were obtained. The positive (negative) part was considered as the production (degradation) term. The reactions with their effective propensities are tabulated in Table 3.

**Table 3:** Input reactions and the associated propensities.

Reaction	Propensities	Description
$\rlap{/}{0}\to {\bf{p}}{\bf{p}}{\bf{E}}{\bf{R}}{{\bf{K}}}_{{\bf{c}}{\bf{y}}{\bf{t}}}$	$\frac{{{\rm{k}}}_{1}{\rm{L}}}{{{\rm{T}}}_{1}}{{\rm{e}}}^{-\frac{{\rm{t}}}{{{\rm{T}}}_{1}}}$	Synthesis and phosphorylation of ${{\rm{ppERK}}}_{{\rm{cyt}}}$
${\bf{p}}{\bf{p}}{\bf{E}}{\bf{R}}{{\bf{K}}}_{{\bf{c}}{\bf{y}}{\bf{t}}}\to \rlap{/}{0}$	$\frac{{{\rm{k}}}_{2}{\rm{L}}}{{{\rm{T}}}_{2}}{{\rm{e}}}^{-\frac{{\rm{t}}}{{{\rm{T}}}_{2}}}$	Degradation and de-phosphorylation of ${{\rm{ppERK}}}_{{\rm{cyt}}}$

### Computation of channel capacity

Cells must respond appropriately depending on the stimuli. The information flows from the extracellular media to the final effectors. This communication process can be is studied using the Information Theory developed by Claude Shannon^23^. One major advantage of Information Theory is that it can be used to characterize and evaluate a signaling pathway^25^. It is considered as a black-box. Information Theory provides some metrics: Channel Capacity, *CC*, and Mutual information, *I*.

These metrics can be used to evaluate how well different input signals are still distinguishable after the signal has been transduced. In a technical setting, this is the limit to which messages can be transmitted reliably. It is important to keep in mind that with the capacity, we can set an upper bound on information transmission.

Under this framework, the quality of the information transmission (mutual information, Eq. (19)) and the maximal amount of recognizable information transmitted through a certain channel (the channel capacity Eq. (20)) can be measured. S represents the input, R is the response elicited by S and H denotes the entropy. All variables are random.

19 $${\rm{I}}\,({\rm{R}};{\rm{S}})={\rm{H}}({\rm{R}})-{\rm{H}}({\rm{R}}|{\rm{S}})=\sum _{{\rm{i}},{\rm{j}}}{\rm{P}}({\rm{R}}={\rm{r}}){\rm{P}}({\rm{S}}={{\rm{s}}}_{{\rm{j}}}|{\rm{R}}={{\rm{r}}}_{{\rm{i}}}){\log }_{2}\frac{{\rm{P}}({\rm{R}}={{\rm{r}}}_{{\rm{i}}}|{\rm{S}}={{\rm{s}}}_{{\rm{j}}})}{{\rm{P}}({\rm{R}}={{\rm{r}}}_{{\rm{i}}})}$$

To characterize the information properties of the FFL, the CC was computed using Eq. (20) to a set of different S and R was measured.

20 $${\rm{CC}}({\rm{R}};{\rm{S}})={{\rm{\max }}}_{{\rm{P}}({\rm{S}})}\,{\rm{I}}({\rm{R}};{\rm{S}})\,{\rm{such}}\,{\rm{that}}\,\{\begin{array}{c}\sum _{{\rm{i}}}{\rm{P}}({{\rm{S}}}_{{\rm{i}}})=1\\ {\rm{P}}({{\rm{S}}}_{{\rm{i}}})\ge 0\end{array}$$

where maximum is taken over all possible input distributions P(s)^28^.

To calculate the CC value, the input probability distributions P(S) must be known. In the biological field this is a limitation, because it cannot be measured the occurrence frequency of a specific stimulus. Nevertheless, we computed the CC using the Arimoto-Blahut algorithm^49^. The computation needs the conditional probability distributions between the inputs and the responses.

Extending the evaluation of the information capabilities for two or more variables, the multivariate mutual information was calculated Eq. (21). This metric settles non-redundant information^24^.

21 $${\rm{MMI}}={\rm{I}}({{\rm{R}}}_{1},\ldots ,{{\rm{R}}}_{{\rm{n}}};{\rm{S}})=\sum _{{\rm{r}}1,\ldots ,{\rm{rn}},{\rm{S}}}{\rm{P}}({{\rm{r}}}_{1},\ldots ,{{\rm{r}}}_{{\rm{n}}},{\rm{s}}){\log }_{2}\frac{{\rm{P}}({{\rm{r}}}_{1},\ldots ,{{\rm{r}}}_{{\rm{n}}},{\rm{s}})}{{\rm{P}}({{\rm{r}}}_{1},\ldots ,{{\rm{r}}}_{{\rm{n}}}){\rm{P}}({\rm{s}})}$$

Probabilistic independence of the variables was assumed. It implies that ${\rm{P}}({{\rm{r}}}_{1},\ldots ,{{\rm{r}}}_{{\rm{n}}},{\rm{s}})={\rm{P}}({{\rm{r}}}_{1}|{\rm{s}})\cdots {\rm{P}}({{\rm{r}}}_{{\rm{n}}}|{\rm{s}}){\rm{P}}({\rm{s}})$. Using the P(S) obtained by the calculation of the CC and the conditional distribution P(r|s) is obtained directly from the data.

### Modeling extrinsic noise

The information transmission through a channel is subjected to noise^26^. The biological noise is originated by two sources: intrinsic and extrinsic. The extrinsic contribution is associated with intercellular fluctuations^18,31^. Particularly, in the signaling pathways, the extrinsic noise is associated principally to variations in the phosphorylation reactions^37,43^. The MAP/ERK pathway model, have an explicit phosphorylation step in the activation of the transcription factor ${\rm{pcFos}}$.

$${{\rm{ppERK}}}_{{\rm{cyt}}}+\mathrm{cFos}\,\mathop{\to }\limits^{{{\rm{k}}}_{11}}{\rm{pcFos}}$$

The parameter k_11_ is the phosphorylation rate. This parameter was transformed into a random variable. It was described by a log-normal distribution. Additionally, two parameters to control the extrinsic noise source were introduced, the intensity $({{\rm{\varepsilon }}}_{{\rm{noise}}}$) and the update frequency (P_ext_). ${{\rm{\varepsilon }}}_{{\rm{noise}}}$ corresponds to the shape of the lognormal probability distribution, and P_ext_ represents the frequency with which k_11_ was sampled (see Fig. 3(a,b)). The scale parameter for the distributions is the nominal value of k_11_ = 0.11; it is reported by Nakakuki *et al*.^7^

### Computational analysis

To solve the time-evolution of stochastic models, we used Gillespie's algorithm^50^. The stochastic simulations were performed for 200 minutes over 1000 realizations. The corresponding histograms were calculated and normalized -by his area under the curve- to approximate it to the conditional probability distributions for every minute of simulation. Therefore, 200-time sets of conditional probability distributions were obtained.

The conditional distributions were used to obtain the CC, one CC value per conditional probability distributions was obtained. To compute the CC, the Arimoto-Blahut algorithm was used^23^. All the algorithms were developed using Python 2.

### Statistical analysis

For this study instead of assuming predefined distributions to compute CC, we developed a stochastic model with the fundamental properties to comprise fundamental properties of studied pathways. Thus, conditional probability distributions were approximated using normalized frequency distributions. We ensured CC accuracy by repeating simulations 1000 times in every experiment to fill properly the response distributions. Consequently, statistical measures like mean and variance are included by taking all the distribution.

## Supplementary information

Supplementary Information
