# Supplementary material for: On Information Extraction and Decoding Mechanisms Improved by Noisy Amplification in Signaling Pathways
Source: Sci Rep. 2019 Oct 7;9:14365. doi: 10.1038/s41598-019-50631-0 (PMC6779762; doi:10.1038/s41598-019-50631-0)
Supplement: Supplementary file 1 — Supplementary Information [file 41598_2019_50631_MOESM1_ESM.docx]

**Supplementary Information**

On Information Extraction and Decoding Mechanisms improved by Noisy Amplification in Signaling Pathways

# Aarón Vázquez Jiménez & Jesús Rodríguez González^*^

Centro de Investigación y de Estudios Avanzados del IPN, Unidad Monterrey, Vía del conocimiento 201, Parque de Investigación e Innovación Tecnológica, 66600 Apodaca NL, México

*VazqAaron@gmail.com, [jrodriguez@cinvestav.mx](mailto:jrodriguez@cinvestav.mx)*

*^*^ Corresponding author*


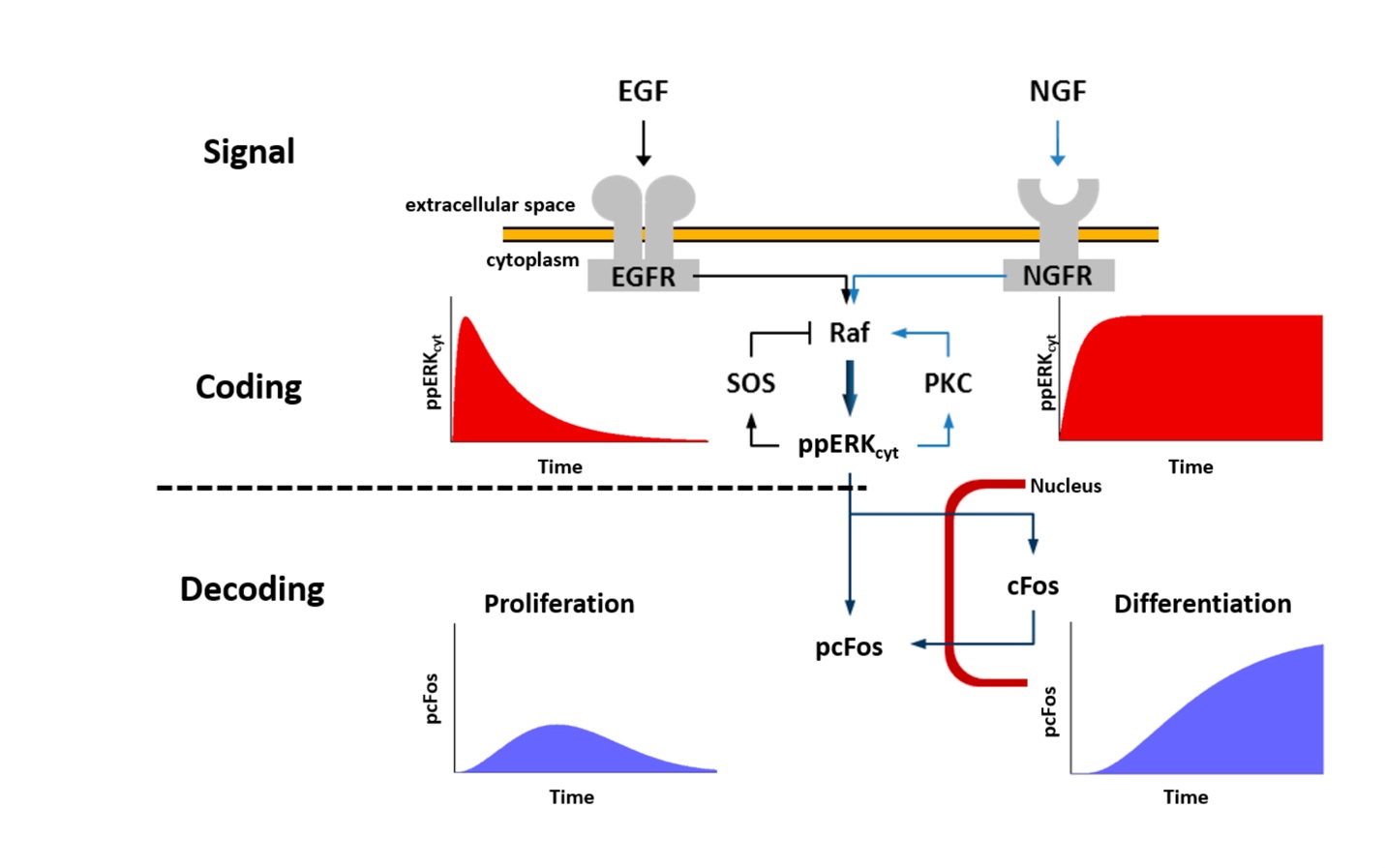


**Figure S1.** The MAP/ERK pathway representation with the encoding and decoding sections summarized.


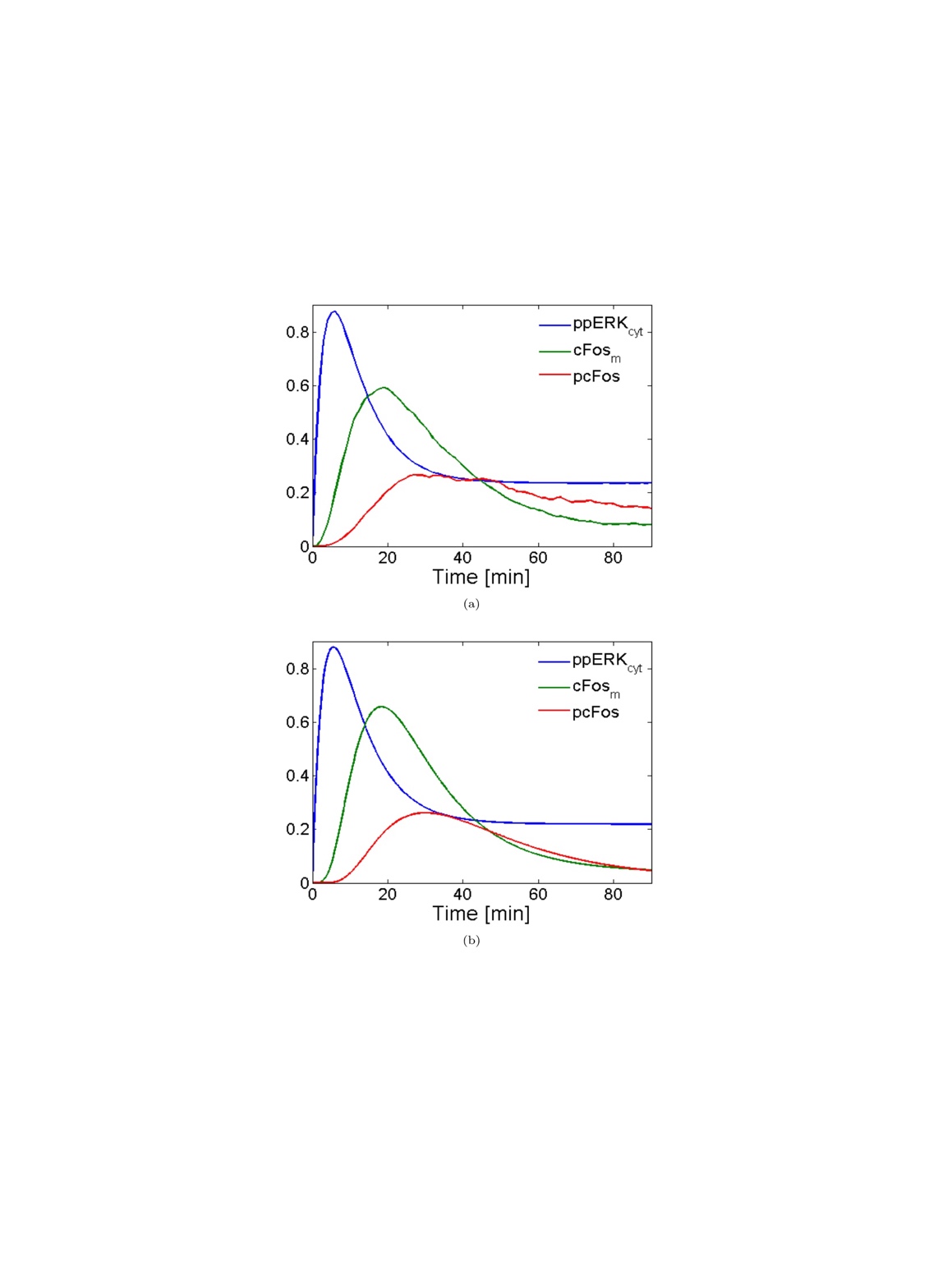


**Figure S2.** Convergence between the stochastic and deterministic models of the MAPK/ERK pathway decoding section, three modelled variables are presented $\text{ppERK}_{\text{cyt}}$, $\text{cFos}_{\text{m}}$ and $\text{pcFos}$. (a) Stochastic model results, the system was solved through the Gillespie’s algorithm, the mean over 1000 realizations are shown. (b) ODE model is given by Eqs. (1)-(10), the model was solved using a fifth order Runge-Kutta solver.

**
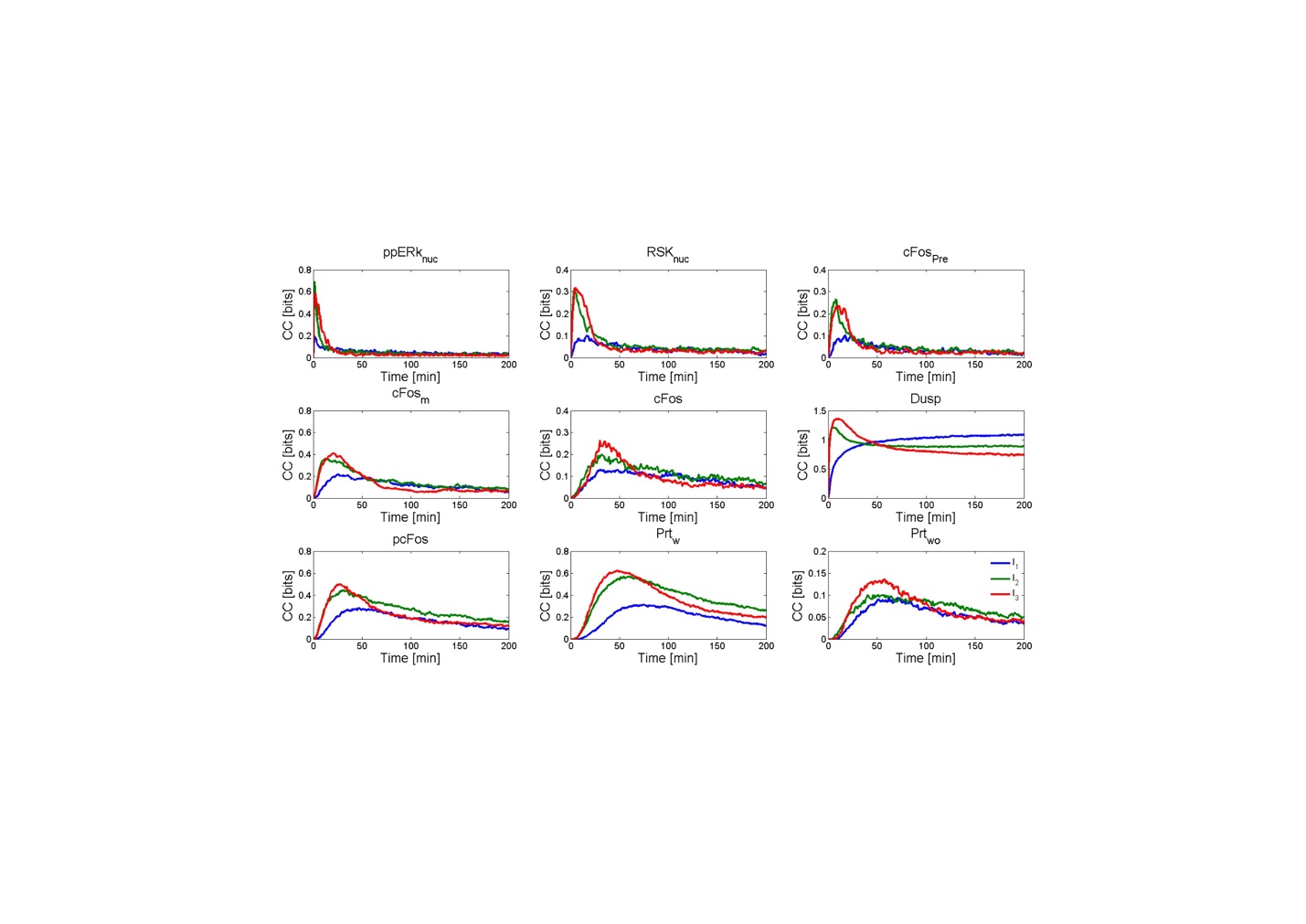
**

**Figure S3.** Channel capacity for all variables of the decoding section of the MAPK/ERK pathway for three inputs: $I_{1}$,$I_{2}$, and $I_{3}$.

**
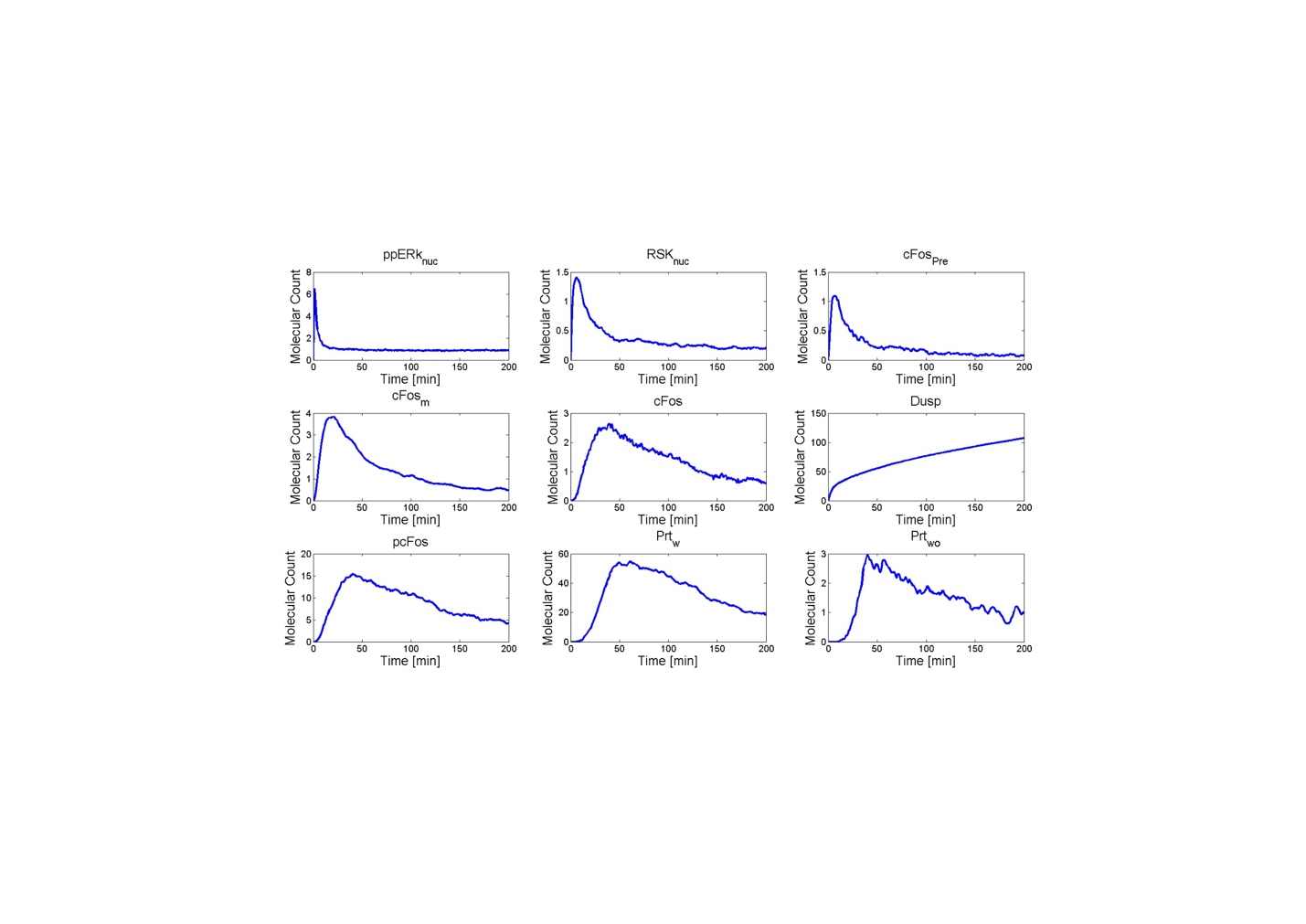
**

**Figure S4.** Temporal dynamics of the molecular count for the pathway variables being stimulated by $I_{2}$ using a scale parameter $\text{L=100}$.

**
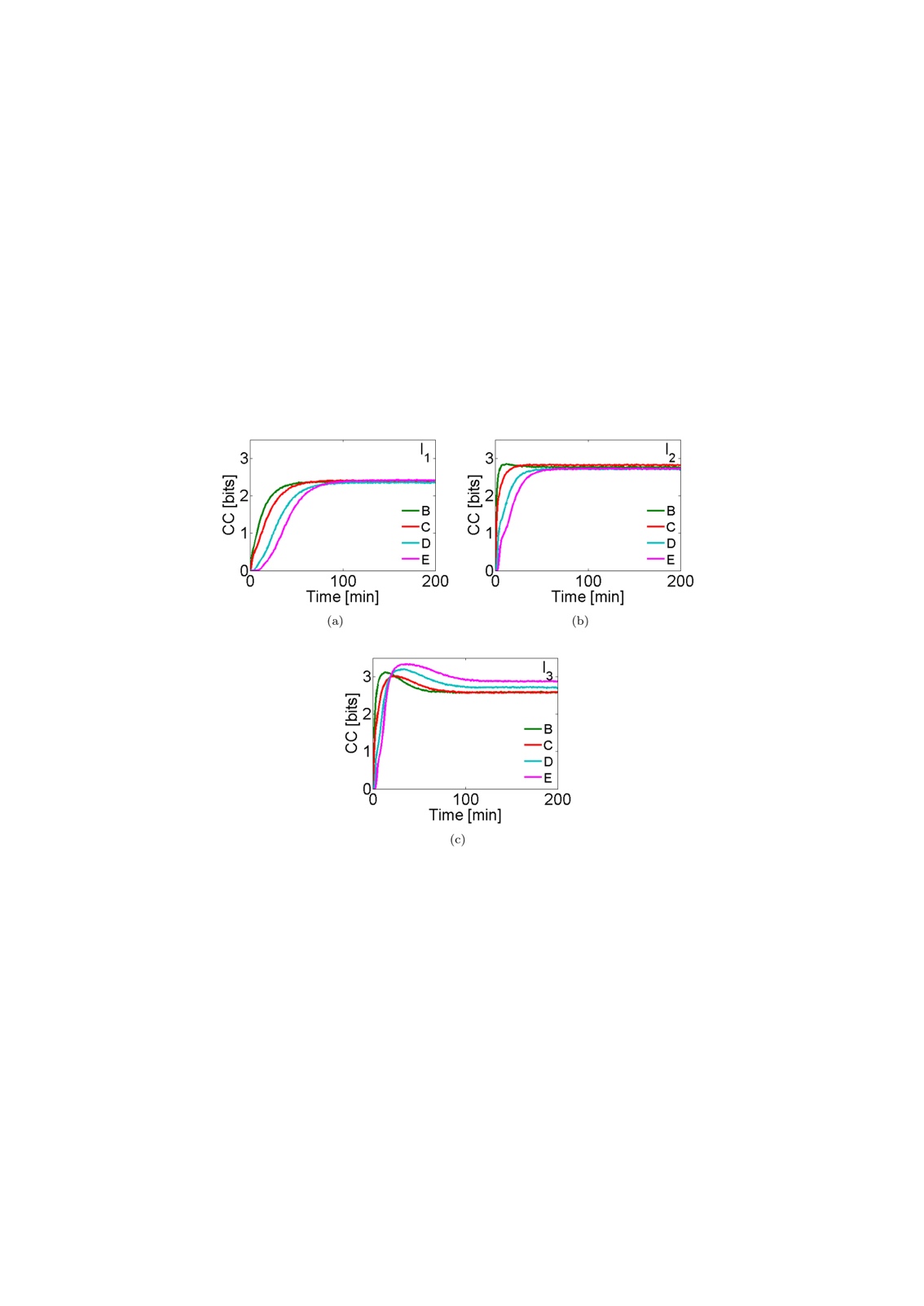
**

**Figure S5.** Channel Capacity for each protein of the minimal decoder model for three different inputs: (a) $I_{1}$, (b) $I_{2}$ and (c) $I_{3}$.


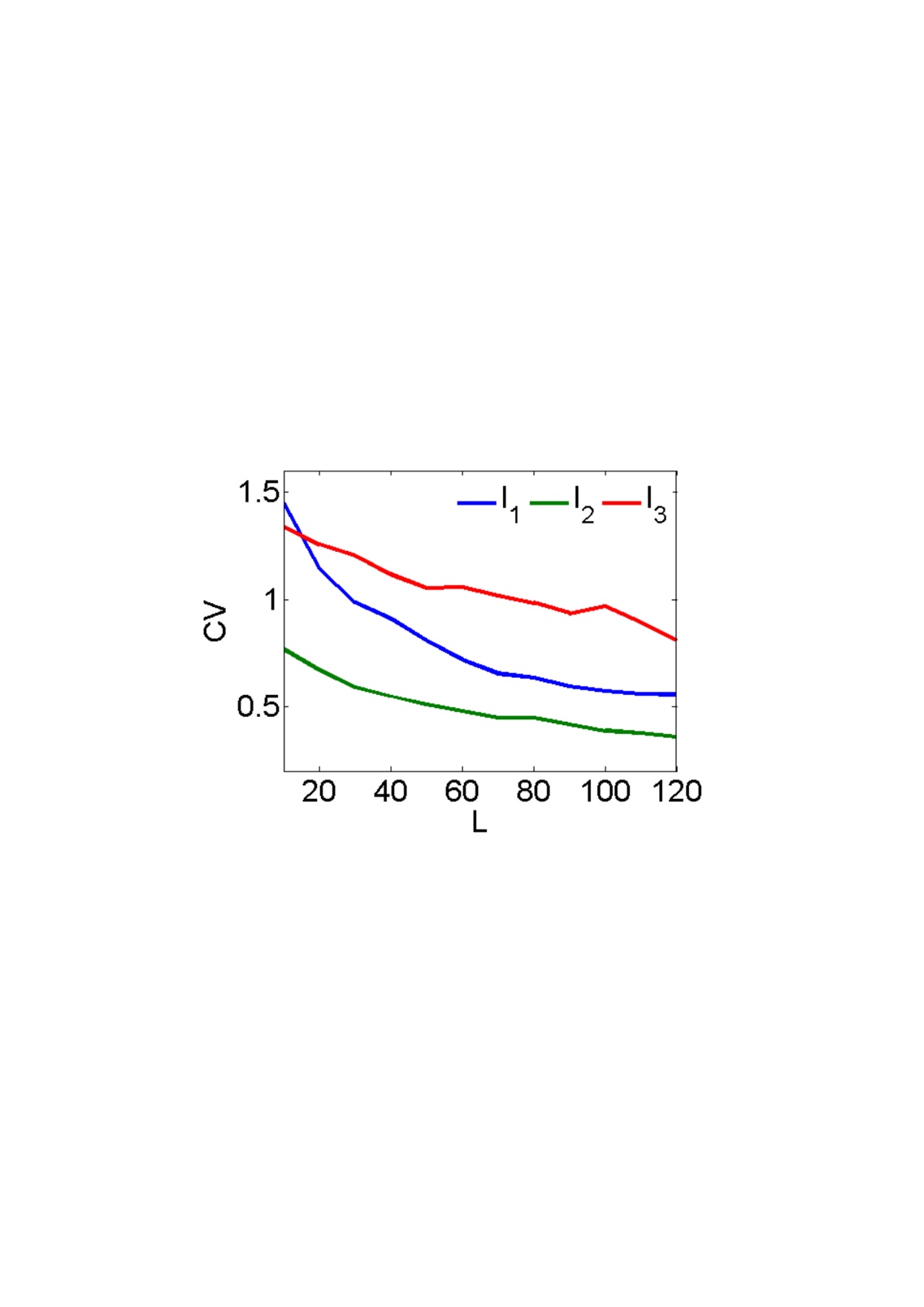


**Figure S6.** The coefficient of variation (CV) versus the scale parameter $\text{L}$ at $\text{t=150 min}$. At this time, the system was considered at steady state. $I_{1}$, $I_{2}$, and $I_{3}$ refer to the three different stimuli. The CV was calculated by the ratio between the standard deviation and the mean over the 1000 realizations. This graph shows the CV to protein E of the minimal decoder model.

**
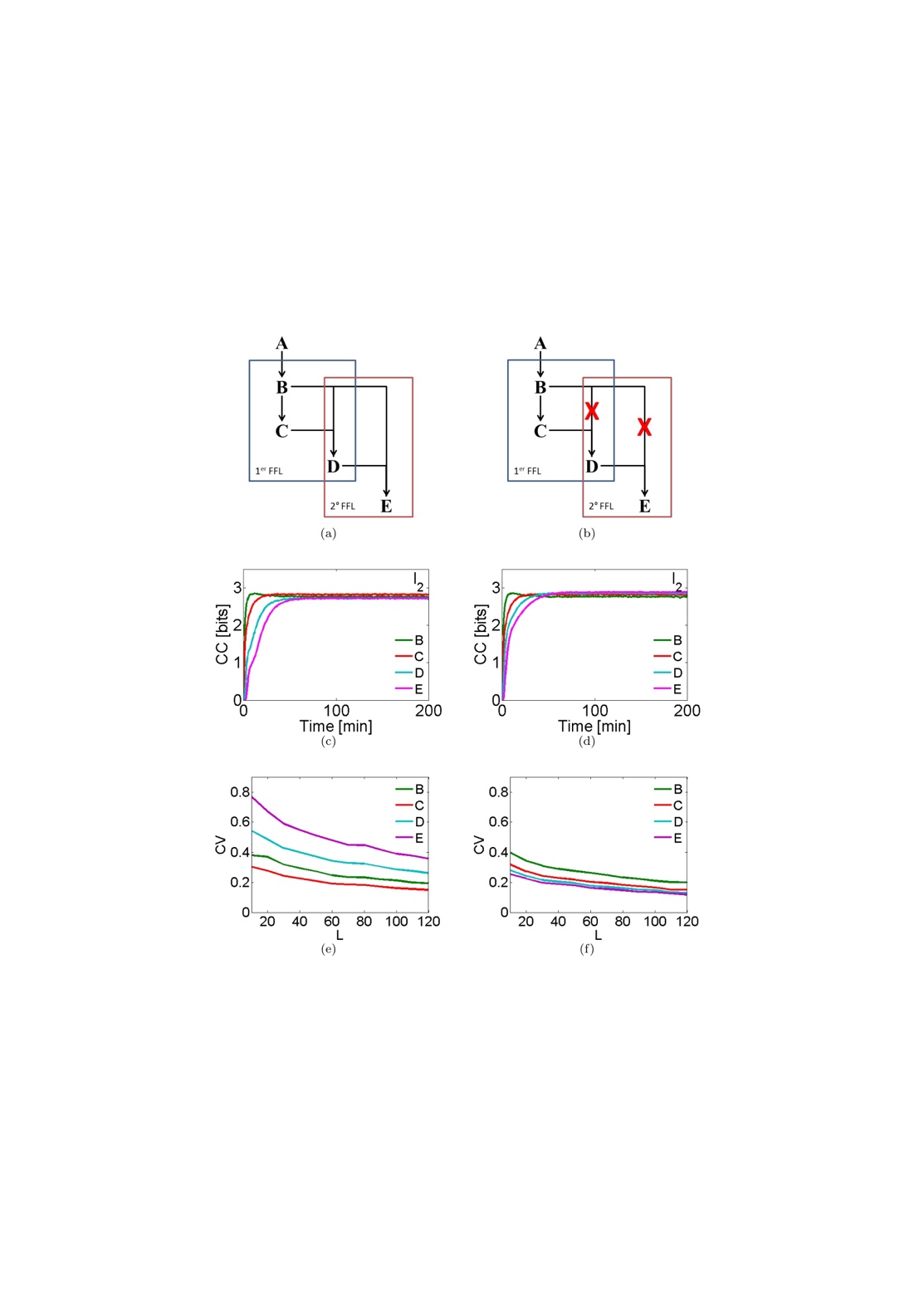
**

**Figure S7.** Comparative study of the minimal decoder model and a consecutive reaction system. (a) Minimal decoder model with two nested FFL. (b) The model with consecutive reactions. (c)-(d) Channel Capacity time series for each variable, respectively (e)-(f) Coefficient of variation for each variable, respectively. Simulation time equal to $\text{200 min}$, for different values of the input scale parameter $\text{L}$.

**
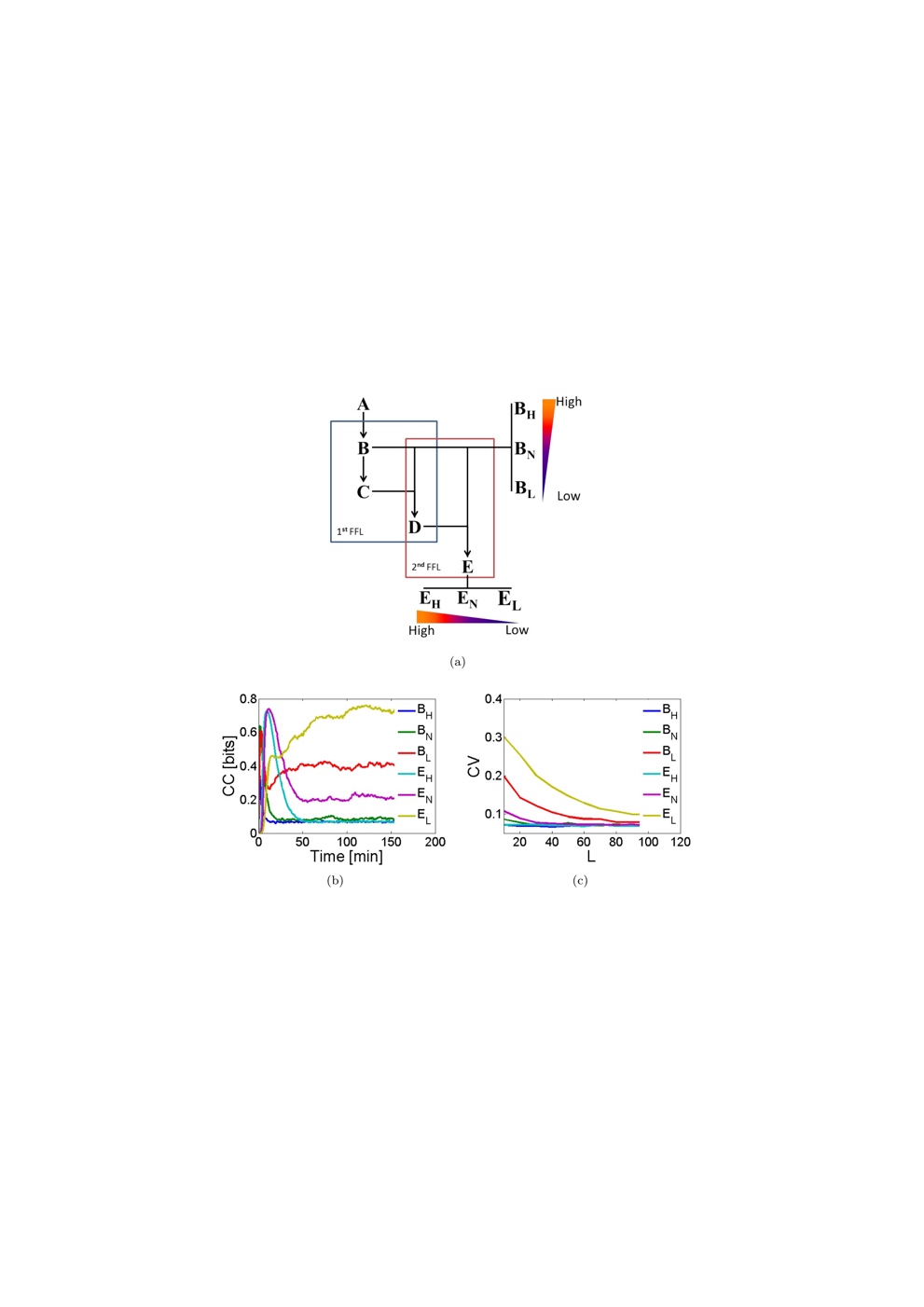
**

**Figure S8.** Affinity exploration for the minimal model. (a) Two gene sets were coupled interacting (W) and without (WO) with the minimal model; the genes with the suffixes **H**, **N,** and **L** are the genes with high, standard and low affinities values, respectively. The change in the affinity value is an order of magnitude up and down to the nominal value. (b) CC for both gene sets over time. (c) Coefficient of variation for each gene set. Simulation time equal to $\text{150 min}$ for different values of the input scale parameter $\text{L}$.

**
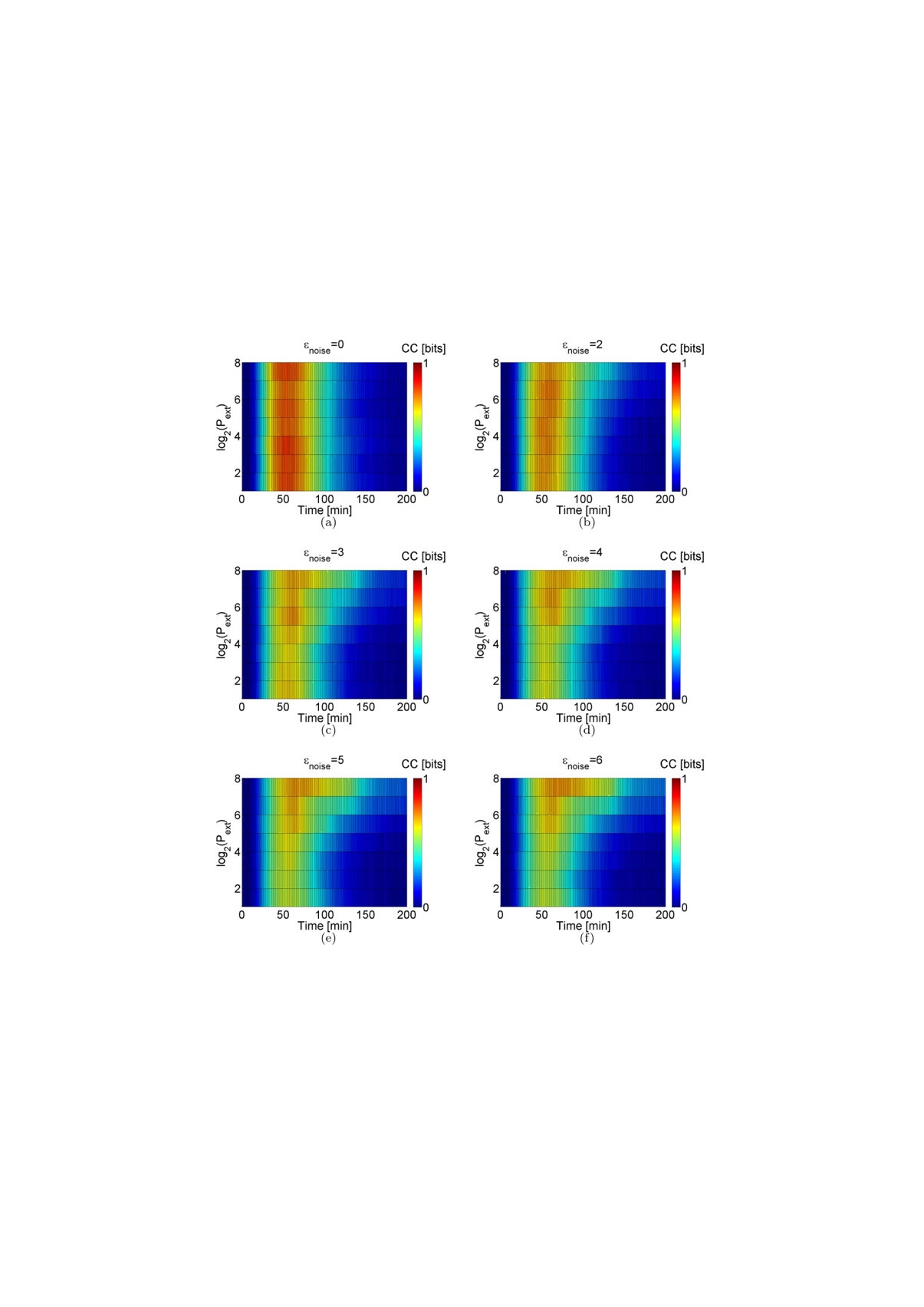
**

**Figure S9.** Channel Capacity to six extrinsic noise intensity values. Simulation time, $\text{t=200 min}$. $P_{\mathrm{ext}}$ represents the update period with which $k_{11}$ was sampled.
